# Enhancing the Effect of Nucleic Acid Vaccines in the Treatment of HPV-Related Cancers: An Overview of Delivery Systems

**DOI:** 10.3390/pathogens11121444

**Published:** 2022-11-30

**Authors:** Ingrid Andrêssa de Moura, Anna Jéssica Duarte Silva, Larissa Silva de Macêdo, Maria da Conceição Viana Invenção, Mylenna Máyra Gois de Sousa, Antonio Carlos de Freitas

**Affiliations:** Laboratory of Molecular Studies and Experimental Therapy—LEMTE, Department of Genetics, Federal University of Pernambuco, Recife 50670-901, Brazil

**Keywords:** carries, cancer, therapeutic, DNA, RNA, papillomavirus

## Abstract

Prophylactic vaccines against human papillomavirus (HPV) have proven efficacy in those who have not been infected by the virus. However, they do not benefit patients with established tumors. Therefore, the development of therapeutic options for HPV-related malignancies is critical. Third-generation vaccines based on nucleic acids are fast and simple approaches to eliciting adaptive immune responses. However, techniques to boost immunogenicity, reduce degradation, and facilitate their capture by immune cells are frequently required. One option to overcome this constraint is to employ delivery systems that allow selective antigen absorption and help modulate the immune response. This review aimed to discuss the influence of these different systems on the response generated by nucleic acid vaccines. The results indicate that delivery systems based on lipids, polymers, and microorganisms such as yeasts can be used to ensure the stability and transport of nucleic acid vaccines to their respective protein synthesis compartments. Thus, in view of the limitations of nucleic acid-based vaccines, it is important to consider the type of delivery system to be used—due to its impact on the immune response and desired final effect.

## 1. Introduction

According to the World Health Organization (WHO), cancer-related diseases represent the world’s second leading cause of death, resulting in approximately 19.3 million new cases and approximately 10 million deaths in 2020 [1]. In addition to genetic factors and habits such as smoking, alcoholism, and poor diet, infectious diseases—especially those arising from viral infections—stand out as responsible for approximately 13% of human cancers [2].

Among viruses, the human papillomavirus (HPV), belonging to the Papillomaviridae family, is linked to squamous cell carcinomas and adenocarcinomas, and is considered the second most prevalent etiologic agent, with 5% of the global burden (approximately 690,000 new cases) [2,3]. Currently, 229 types of HPV are described by the International HPV Reference Center (www.hpvcenter.se; accessed on 1 November 2022) [4,5]. All HPVs can induce benign proliferative lesions (such as warts), but 12 genotypes (HPVs types 16, 18, 31, 33, 35, 39, 45, 51, 52, 56, 58, 59) are considered high-risk because they are capable of inducing malignant transformations [6]. Of these, HPV16 and HPV18 comprise the most oncogenic and prevalent types [7]. Although the distribution of these genotypes varies by geographic area [8], they represent a considerable burden worldwide, especially in developing countries.

These HPVs infect both the cutaneous and mucosal epithelium and are tissue-specific, in which different subtypes preferentially infect each tumor subsite, and there may be specific immune barriers in each microenvironment [9,10]. With a high risk of infection, approximately 80% of people will be infected with HPV at some point in their lives [11]. However, while the immune system usually clears the virus, for some people, the infection remains and can lead to precancerous changes [12]. HPV infection is a major cause of cervical, anogenital, and oropharyngeal neoplasms [13], and more recently, although less prevalently, has been associated with cases of skin cancer [14], lung [15], and esophageal adenocarcinoma (EAC) [16].

Current viral infection control strategies are based on routine screening and prophylactic vaccination, implemented as a worldwide program against low-risk genotypes and the most prevalent oncogenic types [17]. Currently, three different HPV vaccines (Cervarix^®^, Gardasil^®^, and Gardasil^®^-9) have been or are being used worldwide to prevent HPV-related cancers [18]. More recently, the Cecolin vaccine was licensed in China, developed against types 16 and 18 and is currently under review by the WHO [19]. HPV vaccines prevent genital warts, cervical cancer, and most HPV-related cancers, including anogenital cancers [9]. In addition, it reduces the risk of most associated throat and penile cancers [20].

However, progress towards prevention is sometimes discouraging due to the limited access to vaccination and restrictions on screening for HPV-positive malignancies, particularly in developing countries [21,22]. Furthermore, despite the high efficacy linked to a significant reduction in the rate of cervical cancer, acceptance is limited, and they do not benefit patients with established tumors [23]. Traditional treatment options for these individuals, whether in advanced or recurrent stages, include chemotherapy, surgery, and radiation therapy, which are associated with a short survival and substantial adverse effects [24]. As a result, developing therapeutic options for HPV-related malignancies is imperative. Nucleic acid vaccines are safe, quick, and easy to implement platforms capable of evoking efficient adaptive responses [25,26]. However, strategies to increase the immunogenicity of DNA vaccines, minimizing the degradation of mRNA molecules, and enable their acquisition by immune cells are commonly necessary [27]. One way to overcome this limitation is to use delivery systems that allow for the uptake of specific antigens and help modulate the immune response [28].

Approaches based on lipids (e.g., micelles, LNPs, lipoplex, liposomes, and emulsions), polymers (e.g., polymeric nanoparticles, dendrimers, and polymeric nanoemulsions), and microorganisms (e.g., yeasts, bacteria, archaea, viruses, and virosomes) emerge as alternative systems to be explored by presenting chemical components in their external structure that act in different ways as natural adjuvants. Therefore, this review aimed to discuss the influence of these different systems on the response generated by nucleic acid vaccines, evaluating their efficacy, mechanism, routes of administration, advantages, and limitations.

## 2. Therapeutic Vaccines for HPV-Associated Malignancies

Prophylactic vaccines aim to prevent infection by a pathogen, and thus protect the individual from future contact through immunological memory [29]. The application of prophylactic vaccines induces an adaptive immune response primarily focused on the humoral immune response [30]. This immunity is achieved through B cells, but to be successful, it requires the participation of CD4+ T cells, hence depending on effective cellular immunity [31].

Capsid proteins from different high-risk HPV strains are used in preventive HPV vaccinations to generate a neutralizing antibody response that prevents recurrent HPV infection [10]. These vaccines are based on viral capsid protein L1 virus-like particles (VLPs) produced and expressed by yeast (such as *Saccharomyces cerevisiae*) and baculovirus in insect cells [32]. From the spontaneous self-assembly of the L1 protein, a highly immunogenic structure similar to the native conformation of virions is formed, which is then recognized by the immune system cells, inducing the production of neutralizing antibodies [33]. For these vaccines, the significance of the humoral response is evident; nevertheless, studies suggest that B cells may also have an indirect effect in modulating immune responses against HPV-related malignancies [10]. Recent studies show that additional diagnostics and therapies targeting B cells can help predict patients with a better prognosis who would benefit from less invasive treatments [34,35]. In the study by Kim et al. (2020), for example, it was found that in HPV-associated squamous cell carcinomas, B cells improved the overall survival and were activated by radiation and PD-1 blockade. Additionally, Hladíková et al. (2019) found that tumor-infiltrating B cells affect the progression of oropharyngeal squamous cell carcinoma through cell-to-cell interactions with CD8+ T cells.

As for therapeutic vaccines, they require the differentiated modulation of the immune system as both chronic infections and cancers are associated with specific immunosuppression and impairment of the immune surveillance system [36]. This vaccine aims to eliminate the disease by increasing, modulating, or redirecting the immune response, thus forcing the immune system to recognize pathogens and abnormal cells [37,38,39]. Since the pathogenic genesis of HPV is linked to the persistent expression of oncogenic viral proteins, most of them are considered as therapeutic targets against HPV-related malignancies [23].

It is currently recognized that the oncogenic causative role of high-risk HPV types is fundamentally attributed to the action of the main viral oncoproteins, E6 and E7, which, respectively, inhibit the tumor suppressors p53 and pRB, which are involved in the development of malignancies [40]. These oncoproteins act synergistically, targeting various cellular pathways involved in regulating cell cycle control, promoting cellular immortalization, and facilitating invasion and malignant progression in the host [41]. In addition to these, the HPV E5 protein has been considered an attractive therapeutic target to prevent the progression of precancerous lesions into invasive cervical cancer since it is considered a putative oncogene that acts in the first stage of carcinogenesis, is responsible for regulating the MHC-I, and mediates immune evasion [23,42]. Oncoproteins are constitutively produced at high levels in tumor cells and are not found elsewhere in the human body, which distinguishes HPV-associated cancers [41,43]. This constitutive production makes them an excellent target for the therapeutic vaccines designed to provoke a specific antitumor response, targeting cells that express the antigens and limiting the danger of harming healthy tissue [44].

Unlike prophylactic ones, they are primarily focused on cell-mediated immunity and involve the interaction between APCs and naive T cells that will become CD4+ effector cells (via MHC-II) or CD8+ (via MHC-I) [45]. CD4+ cells differentiate into helper T cells that secrete effector molecules, such as cytokines, and may act by increasing the CTL (cytotoxic T lymphocyte) immune response, activating antibody-producing B cells, and modulating regulatory or inflammatory profiles [23]. As with cancer vaccines, CD4+ and CD8+ T cells travel to the tumor site and, upon finding corresponding antigens, kill tumor cells by cytotoxicity and cytokine production [45]. Therapeutic vaccine platforms against HPV-associated cancers in clinical and preclinical stages include bacterial and viral vectors, peptides, proteins, nucleic acids, and, more recently, whole cell-based vaccines [43,46]. Most platforms consisting of attenuated or inactivated pathogens generate a sufficient signal to the immune system to produce memory cells and antibodies [47]. However, they present problems associated with safety, such as virulence reversal, limitations related to efficacy against rapidly evolving pathogens, and demand for production systems with high levels of biosafety [48]. Nucleic acid vaccines, on the other hand, are third-generation vaccines that focus on the synthesis of an antigen of interest and presentation by MHC molecules, allowing the specific induction of cellular responses, which are important in the treatment of cancer, allergies, and autoimmune diseases [49,50]. Furthermore, the production of nucleic acid-based vaccines makes them quick to develop since there is no large-scale growth of highly pathogenic organisms, reducing the cost and the risk of contamination and infection [50].

## 3. Nucleic Acid Vaccines against HPV-Related Cancers and Their Limitations

**Figure 1:**
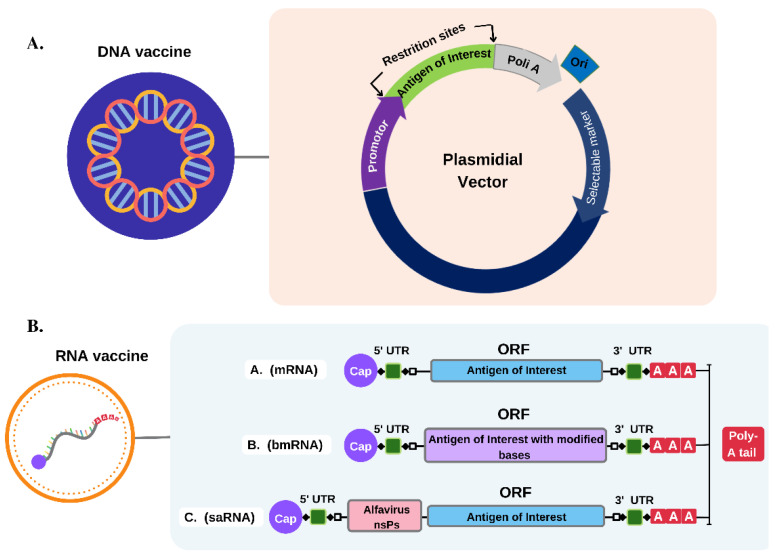
Structural composition of nucleic acid vaccines. (**A**). The DNA vaccine is formed by a modified plasmid that has in its composition a sequence referring to the gene of interest, after the promoter region, as well as the marker gene for resistance to certain antibiotics. (**B**). The RNA vaccine is formed from a single-stranded RNA molecule that can be constructed in three ways, mRNA containing Cap 5′ UTR, the gene of interest and 3′ UTR Poly-A tail; bmRNA containing Cap 5′ UTR, gene of interest with modified bases and 3′ UTR Poly-A tail; and saRNA containing 5′ Cap UTR, alphavirus nsPs followed by the sequence of interest and 3′ UTR Poly-A tail.

The DNA vaccine can evoke efficient cellular, and humoral responses compared to conventional and protein-based vaccines and is considered safer, more stable, and easier to manufacture [25].This vaccine is generally produced using recombinant DNA technology and consists of a plasmid containing one or more genes that encode the vaccine antigen (Figure 1A) [51]. Consequently, it is designed to increase the translation and insertion of DNA into cells so that the activation of CD4+ T cells, better induction of CTLs, and antibody production by B cells occurs [51]. After its internalization by the cell and entry into the nucleus, the DNA is transcribed and then translated in the cytoplasm [52]. Then, the encoded antigen is expressed and presented by the MHC to generate CD4+ and CD8+ T cell activation and the indirect activation of humoral immunity [53,54].

It is important to note that, although there is still an emphasis on a potential for genotoxicity by chromosomal integration, studies have already shown that it is highly unlikely that the vector will integrate into genomic DNA [55,56,57]. The first DNA-based vaccine for emergency use, in humans, was developed in India for COVID-19. It is known as ZyCoV-D, and it produces the spike protein of the SARS-CoV-2 virus, inducing immune responses aimed at viral elimination. Interim findings from the phase III clinical trial revealed robust immunogenicity, tolerance, and safety profiles [58]. Although this was a milestone for the clinical use of the DNA vaccine, despite veterinary clearance against infectious agents from fish, companion animals, and farms, this platform is still not approved by the FDA for use in humans [59]. One of the main limitations is DNA delivery. These bottlenecks are frequently caused by low-protein expression levels and poor APC uptake, resulting in decreased immunogenicity and low transfection rates [51,60].

Examples of clinical trials of therapeutic DNA vaccines against HPV-associated malignancies can be viewed in Table 1. Overall, these vaccines were well tolerated without severe adverse effects and could induce detectable humoral and T-cell responses. However, the percentage of regression or cure was modest, and they are not yet licensed. The phase II vaccine GX-188E, for example, composed of E6/E7 pDNA, was applied to patients positive for HPV 16/18 with CIN3. Among these, 67% of patients 36 weeks after the first injection showed histopathological regression. Although these patients showed increased HPV-specific IFNγ responses, it was impossible to directly correlate with regression [61]. Another vaccine, however, called VGX-3100, developed by INOVIO Pharmaceuticals, reached Phase III of clinical trials (REVEAL 1 and REVEAL 2) and consisted of a mixture of two plasmids containing the optimized E6 and E7 genes of HPV 16/18 delivered by electroporation. This immunotherapy is focused on the treatment of high-grade precancerous cervical dysplasia. Nonetheless, no study results have been published in Clinical Trials (NCT03185013) and are only available as a press release [62].

Like the DNA vaccine, the RNA vaccine is a safe and effective platform that encodes antigenic proteins and induces an immune response via antigen presentation [26]. To date, three types of RNA vaccines have been developed, including conventional non-amplifying messenger RNA (mRNA) molecules; self-amplifying mRNA (saRNA) vaccines; and base-modified conventional non-amplifying mRNA (bmRNA) [69]. The basic structure of these vaccines consists of the conventional arrangement of coding molecules present in the body, presenting a 5′ CAP, UTRs regions, open reading frame (ORF), and a PolyA tail (Figure 1B) [70]. 

Although its application has been previously restricted by RNA instability and inefficient delivery, several studies have investigated this platform for prophylactic and therapeutic applications [71]. Recently, mRNA vaccines developed by Moderna and Pfizer/BioNTech were licensed against COVID-19, reaffirming the potential of this long-studied platform [72]. In mRNA and saRNA vaccines, the property of stimulating adaptive immunity with the induction of B cells and CD4+ and CD8+ T cells was verified [73]. In contrast to the DNA vaccine, RNA vaccines do not require targeting and entry into the nucleus, as their translation into antigenic proteins occurs in the cytoplasm [74]. After processing, epitopes from these proteins are presented via MCH-I by cross-presentation with APCs, which results in the activation of CD8+ T cells and the induction of the immune response [48]. In the case of exogenous proteins, these can be taken up by APCs, degraded by endosomes and presented via MHC-II, leading to the induction of CD4+ T cells [75]. Recently, Komdeur et al. (2021) published the results of the Phase I clinical trial of the first human RNA vaccine based on the Semliki Forest virus (SFV) encoding HPV 16 E6 and E7 antigens against HPV-related cancers. Immunization was safe and well tolerated, resulting in positive vaccine-induced CD4+ and CD8+ T cell-specific immune responses in all study patients with a history of CIN [68]. In addition, the anti-CD40 RNA vaccine (BNT113) developed by Pfizer/BioNTech is currently recruiting previous and recurrent HPV16+ patients against major HPV-associated cancers in its phase I clinical trial [67].

However, mRNA vaccines also have limitations. These are mainly related to low stability due to degradation and delivery optimization problems, since specialized delivery systems are needed that allow the integrity of the genetic material and its entry into cells [76,77]. In addition, although they are also considered easy to manufacture and safe, there are logistical limitations regarding the distribution of these vaccines due to the requirement of a cold chain [78,79]. Once both DNA and RNA vaccines have limitations regarding their therapeutic effects, such as a modest percentage of regression or cure, a low correlation between tumor regression and IFNγ responses, and limited thermostability [61,76,77], some strategies to improve T cell function may be necessary to achieve optimal immune responses [36,39]. Among these optimizations are, for example, constructs containing synthetic antigens with multiple epitopes for TC8+, TCD4+, or both, the optimization of codons in the sequences of DNA and RNA, in addition to the use of fat-soluble delivery systems, and platforms with natural adjuvants in the cell wall [80]. The next topic addresses delivery systems used for nucleic acid vaccines that could be further employed in future HPV vaccine strategies.

## 4. DNA/RNA Vaccine Delivery Systems: A Path to Improve Immunogenicity

Measures that aid or optimize vaccine formulation are increasingly being researched in order to overcome the limitations of nucleic acid vaccines [27]. For DNA vaccines, the most common tactics to increase immunogenicity include codon optimization, electroporation as a physical delivery method, and adding CpG motifs [70,81]. In addition, the use of molecules and systems capable of helping to modulate the immune system as well as facilitating the uptake by antigen-presenting cells (APCs) is increasingly necessary to generate a robust and well-targeted response [51].

Adjuvants are compounds co-injected with the antigen that improve or shape the immune response, aiming to increase the immunogenicity of vaccines, reduce the number of doses needed, and extend immunological memory [31,82]. This adjuvant action is accomplished through mechanisms associated with the activation of the innate immune system that relates to the uptake, presentation, induction of inflammatory mediators, and adjustment of costimulatory molecules [27,36,83]. These substances should ideally have stability and compatibility with the antigen, safety, low cost, and be susceptible to degradation [84]. Adjuvants currently incorporated into FDA-approved prophylactic vaccines or clinical-phase therapeutics that primarily include aluminum-based substances such as aluminum phosphate and aluminum hydroxide, and AS04 (which combines aluminum hydroxide with the monophosphoryl lipid A-MPL agonist) [85]. However, several other adjuvants have been developed and approved for use in licensed human vaccines. In addition to those formulated with aluminum salts, Imiquimod (R837), RIBI-529, oil-in-water emulsions (AS03, AF03, and MF59), and Toll-like receptor (TLR) agonist substances such as TLR9-based agonists are used in synthetic DNA sequences (CpG 1018), consisting of MPL (TLR4 agonist) and QS-21, as the adjuvant AS01B [70,85,86].

In the front line of defense against a pathogen, the innate immune system provides a nonspecific response mediated by cells (phagocytic, dendritic, natural killers (NKs)) and by complement molecules through the recognition of molecular patterns by Pattern recognition receptors (PRRs), such as Toll-like (TLRs), NOD (NLRs), RIG-I (RLRs), and C-type lectins [87,88]. Many adjuvants can directly or indirectly activate these receptors to stimulate different types of innate immune responses that, if bound to antigens, can initiate and potentiate areas of the adaptive immune system (composed of T and B lymphocytes, NKs, and mediate molecules such as cytokines [89]. The activation of this system by antigen recognition through the immune synapse with costimulatory molecules and MHC allows the inactivation of the pathogen and the development of immunological memory [90]. It is able to affect the immune response by balancing the induction of humoral and cellular responses. This is made possible by the way in which CD4+ T cells are activated and the types of cytokines released, which results in the expansion of subsets of helper cells (such as Th1, Th2, Th17 and Treg) [91].

By secreting antibodies and activating granulocytes (such as neutrophils and eosinophils), Th2 cells mediate the activation and maintenance of the humoral immune response, necessary to remove extracellular infections such as parasites and allergens [92]. The Th1 response, on the other hand, is involved in establishing cellular immunity against intracellular pathogens and cancer cells. Interestingly, individuals with cervical cancer tend to have a Th2 response rather than a Th1 response, and changes in cytokines that drive a Th2 response have also been discovered in cervical tumors, but this has not been fully investigated in HNSCC HPV positive [10,93]. A Th1 response, on the other hand, has been linked to better outcomes in oropharyngeal squamous cell cancer [94,95]. In addition to these, an anti-inflammatory subset of CD4+ T cells, called regulatory T cells (Tregs), is linked to immune homeostasis, promoting self-tolerance [96]. Tregs help prevent autoimmune diseases, allergies, and some types of cancers by ensuring that the immune system’s reaction to self and foreign antigens is balanced [97]. Moreover, Tregs were found in significant numbers in cervical intraepithelial neoplasia (CIN) and cervical cancer, and their frequency corresponded to disease severity, indicating that Tregs may be involved in interfering with anti-HPV immunity [10,98,99]. Furthermore, FoxP3+ regulatory T cell infiltration is a strong and independent prognostic factor in head and neck squamous cell carcinoma [100]. Therefore, to produce a vaccine with the potential to increase the immune response, in addition to considering the parameters that affect its potency and efficacy, it is also necessary to add appropriate adjuvants to its composition and amplify the stimulation of the immune system [101].

Adjuvants are generally classified according to the mechanism of action as immunomodulatory molecules, delivery systems, or integrated compositions [88,102]. Immunomodulatory adjuvants, mainly represented by cytokines, chemokines, Toll-like receptor agonists and antimicrobial peptides, act by increasing the host’s immune response [82]. In nucleic acid vaccines, there is a certain limitation in the number of clinical and preclinical studies using immunomodulators in their formulation, especially in mRNA vaccines. This may be reflected by the auto-adjuvanticity of the genetic material that leads to specific humoral and cellular immune responses due to the inherent ability of PRRs to activate the innate immune system as well as the nature of the vaccine [103].

In the DNA vaccine, plasmid DNA is intrinsically adjuvanted by the recognition of receptors such as TLR9, NALP3, and cGAS [104]. Although its general use as an adjuvant has limitations, DNA vaccination induces a more Th1-biased immune response and activates MHC antigen presentation pathways for inducing CD8+ and CD4+ T cell immune responses. The strategies of the co-formulation of adjuvants on DNA plasmids encoding the gene of interest, or co-immunization, have been tested in a few studies, but have been used to improve and increase vaccine potency [51]. Among the interleukins, IL-12 has been the most used in therapeutic vaccines against HPV-related cancers, and in general, it appears to improve the efficacy aspects of the DNA vaccine by helping to produce specific humoral and T-cell responses [63]. This pro-inflammatory cytokine has several effects, including the regulation of NK cells and activation of T cells, resulting in a protective Th1 response that stimulates IFN production by T lymphocytes [105]. As for mRNA, it can be recognized by Toll-like receptors such as TLR3, TLR7, TLR8, TLR13, and gene-like receptors I (RIG-I), and is associated with MDA-5 melanoma differentiation [106].

However, because RNA is highly immunogenic and prone to degradation, modifications were made that ultimately extended its lifespan but also reduced its modulation of immune responses via cytoplasmic receptors [107]. Although there have not been any clinical and preclinical trials in the literature to date using immunomodulatory molecules for this vaccine, current research aims to increase stability and modulate the immune response to achieve different levels of immunogenicity and antigen expression through modifications of nucleosides and mRNA components (such as *5′* CAP) [106,108]. One such modification was the switch from uridine to pseudouridine, which increased mRNA stability and base methylation to reduce recognition by TLRs, which facilitated its use as a vaccine platform [109,110]. However, depending on the desired vaccine outcomes, it is crucial to balance the resulting adjuvant effects and translation activity to achieve optimal immune responses [111].

The incorporation of immunoadjuvants into nucleic acid vaccines represents a promising approach to enable the controlled induction of the innate immune system [111]. However, in addition to knowing the profile of these immunomodulators, it is important to take into consideration how the antigens will be delivered and presented to the cells of the immune system. Thus, another strategy for such a vaccine is the use of carrier or integrated adjuvant delivery systems to increase transfection efficiency, ensure the integrity of the genetic material, and provide a better recognition of immune system cells [112]. Many human vaccines, whether in in vitro or preclinical and clinical research phases, use materials such as liposomes, lipid nanoparticles, aluminum salts, emulsions, and virosomes as delivery platforms for the vaccine antigens [113]. In addition to these, other systems based on microorganisms, lipids, polymers, inorganic molecules, and nucleic acids are investigated as carrier particles [114]. Once injected and containing the antigen of interest, these systems can damage or kill the host cells at the injection site, thus triggering responses against the compounds released by the cells [115].

Vaccine delivery systems can function as antigen-associated carriers with adjustable release as well as immune cell recruiters owing to the production of a pro-inflammatory reaction at the injection site, facilitating antigen-specific uptake [116,117,118]. Given the vast diversity of systems, each with its unique properties, factors such as hydrophobicity, size, charge, surface modifications, and material type can influence the interaction with nucleic acids and how they will be perceived by immune cells after vaccine inoculation [119]. Thus, considering the physical and immunological differences of the different carriers for nucleic acid vaccines, the main lipid, polymer, and microorganism-based systems used in nucleic acid vaccines are addressed below (Table 2).

### 4.1. Lipid-Based Delivery Systems

Lipid nanoparticles (LNPs) are an intensively researched non-viral vector for the in vivo delivery of nucleic acid vaccines [142]. NPs refer to all nanoparticles composed of lipids, including various classes such as liposomes, lipoplexes, nanostructured lipid carriers, and cationic nanoemulsions [143]. LNPs are increasingly being used in immunotherapy against cancers and infectious diseases and require specific types and ratios of lipid components when used in nucleic acid vaccines [119].

#### 4.1.1. Liposomes

Liposomes were the first nucleic acid carrier particles to be FDA-approved (with the Doxil Liposome) and the first nanomedicine delivery platform to advance from proof-of-concept to clinical application [144]. They consist of amphiphilic spherical particles composed of one or more phospholipid bilayers surrounded by an aqueous core in which the materials of choice can be encapsulated [119]. In their composition, there is an interaction between the hydrophilic (polar head) and hydrophobic (apolar tail) parts, forming vesicles [145]. They are biodegradable compounds that are versatile, easy to formulate, have substantial efficacy, and may exhibit minimal toxicity depending on the nature of their components [119,146].

Although in vivo studies still evaluate the transport efficiency of nucleic acids in pure form, the most common liposomal forms today are hybridized with adjuvant molecules, targeting ligands and polymers [147]. More recent studies of nucleic acid vaccines with conjugated liposomes have shown promise against infectious diseases and cancer immunotherapy and point to aiding in the induction of cellular and humoral responses (Table 2). In addition, the size of liposomes is known to affect the induction of the vaccine response. In general, small liposomes induce a more polarized Th2 response and are therefore more targeted in prophylactic vaccines [148]. On the other hand, larger liposomes help in Th1 bias, being targeted in therapeutic vaccines for cellular response formation [101].

Because nucleic acids are anionic, liposomes containing cationic lipids such as DOTMA (N-[1-(2,3-dioleoyloxy) propyl chloride]-N,N,N-trimethylammonium), DOTAP (1,2-dioleoyloxy-3-trimethylammonium), and DDA (dimethyldioctadecylammonium) are preferred [120,122,149]. These lipids, by possessing a positive charge, interact electrostatically with the negative charge of nucleic acids, allowing stability in the encapsulation of the genetic material in the aqueous core [149]. Additionally, they possess adjuvant effects that can be modulated by the nature of the cationic molecule, stimulating the innate immune response, pro-inflammatory mediators, and cytokines [150]. On the other hand, DOPE (1,2-dioleoyl-sn-glycero-3-phosphoethanolamine), which is also widely used, is a neutrally charged auxiliary lipid used to favor cell membrane destabilization and aid the endosomal escape of nucleic acids into the cytoplasm [121,146]. However, destabilization by serum proteins when delivered intravenously, charge-dependent, cytotoxicity and rapid elimination from the body make the transition of liposomes to clinical research problematic [123].

#### 4.1.2. Lipoplexes

Lipoplexes are liposome-based formulations that are formed upon the electrostatic interaction between the liposome cation and the anionic part of the genetic material [151]. These formulations are characterized by their poor encapsulation, low tolerability, and tendency to aggregate and not completely release the nucleic acid into the target cell [152]. Although the existence of lipoplexes in the drug Patisiran has provided a milestone in the development of siRNA-based drugs, due to these impediments, there are few preclinical and clinical studies employing this system [153]. As with liposomes, some researchers create hybrid formulations with polymeric particles, forming lipopoliplexes (LPPs) and varying their loading to improve the safety and retention in the desired organs after injection [125]. On the other hand, Peletta et al. (2021) developed a DNA vaccine delivered by cationic lipoplexes against SARS-CoV-2. In this study, it was found that lipoplexes amplified in promoting antibody induction and the generation of balanced Th1/Th2 responses in mice against SARS-CoV-2, comparable to those obtained by electroporation with naked DNA. Already in the field of immunotherapy, small-interference RNAs (siRNA) and their interaction with pH-sensitive lipoplexes, peptides, and cell-penetrating polymers are being studied for better uptake by tumor cells [152].

#### 4.1.3. Emulsions and Cationic Nanoemulsions

Oil-in-water adjuvant emulsions contain MF59, AS03, and AF03. However, they are utilized prevalently with protein and subunit vaccines and are infrequently employed in nucleic acid vaccines. In the literature, it is reported that MF59 has inclinations towards Th2 immune responses, the stimulation of the humoral and cellular responses, and low toxicity [154]. However, because it was not directly employed as a delivery method for the DNA vaccine, it was not feasible to see a direct association with the reaction created by the vaccination. As for cationic nanoemulsions, they are colloidal particles consisting of an oily core in an aqueous phase stabilized by a cationic lipid or a combination of PEGylated lipids or phospholipids [155]. These nanoemulsions have the potential to inhibit particle aggregation, produce stability, and enhance endosomal escape [156]. However, due to the existence of positive charges and their potential toxicity, the inclusion of non-ionic surfactants such as those employed in the formulation of MF59 (Tween and Span) is advised [156]. In addition, they are examined as carriers for gene-editing treatment and in delivery for protein expression by nucleic acids in immunotherapy of mucosal disorders [127,157]. In the study of Schuh et al. [127], for example, a nasal injection of nanoemulsions carrying a DNA plasmid targeting IDUA protein expression (pIDUA) was undertaken as an attempt to target the brain aiming at gene therapy of Mucopolysaccharidosis Type I.

#### 4.1.4. Lipid Nanoparticles

Lipid nanoparticles are the most widely used lipid-based systems in nucleic acid vaccines. Although LNPs also encompass other types of lipid systems and have similar composition, they are in themselves a type of system as they exhibit a different organization [158]. Unlike liposomes that contain an aqueous core surrounded by a bilayer, lipid nanoparticles can contain lipids in the core as well as other particles such as ionizable lipids and PEGylates [147]. LNPs have gained even more prominence recently due to their use in Moderna and Pfizer’s mRNA vaccines against COVID-19 [128,129]. Furthermore, in the DNA vaccine field, a study developed by Mucker et al. (2020) showed that LNPs, commonly used in RNA vaccines, also provided improved stability to DNA and the induction of cellular responses for immunoprophylaxis in animals. In approved COVID-19 vaccines, LNPs consist of an ionizable cationic lipid to facilitate internalization, a PEG-lipid to prevent aggregation, cholesterol, and a neutral phospholipid that contributes structurally [159]. Both vaccine types generate significant neutralizing antibody titers and robust antigen-specific CD8+ and Th1-like CD4+ T-cell responses [120,160]. In these vaccines, LNPs provide delivery and protection against degradation by ribonucleases and generate the activation of the innate immune response by generating transient local inflammation at the injection site [161]. However, they need to be stored at low temperatures, requiring a cold chain along the distribution [162]. Moreover, because they contain a certain amount of water in the core, there is the possibility of decreased efficacy and instability due to the possible hydrolysis of the mRNA [161]. An alternative is the use of solid lipid nanoparticles, which can be lyophilized, improving the large-scale distribution of the vaccine, and promoting lower chances of hydrolysis [163].

### 4.2. Polymer-Based Delivery Systems

Like lipidic nanoparticles, polymeric systems present a great diversity of molecules and are widely used in drug formulation and gene therapy [164]. The polymer-based systems can be divided in terms of origin as: natural and synthetic polymers, and in terms of type: polyamino acids, polysaccharides, polyamines, polyamidoamines, and polyesters [114]. In recent years, great progress has been made in the employment of polymeric materials as protein carriers and third-generation vaccines [115]. Although they have fewer clinical studies than other systems—especially for mRNA vaccines—these polymers have similar benefits, including stability, diversity, targeting, immunomodulatory activity, and personalization [114]. Cationic polymeric nanoparticles, polyplexes, micelles, lipopolyplexes, copolymers, and dendrimers are examples of representative polymeric formulations used for nucleic acid vaccine delivery (Table 2). However, as with LNPs, problems such as charge-induced cytotoxicity can lead to unwanted side effects, and so the PEGylation of the material is performed to increase stability and decrease overall charge, or the use of neutral polymers [165]. The problem is that this PEGylated material may go on to generate antibodies specific to the delivery system after administration, as occurs with microorganism-based delivery systems [166]. Neutral polymers, on the other hand, are less stable and offer the less efficient transfection of the nucleic acid [164]. In addition, factors such as biodegradability, molecular weight, loading, aggregation, hemolysis, and suboptimal endosomal escape need to be considered and optimized for assembling a successful polymer delivery system [167].

#### 4.2.1. Polyplexes

When polymers are complexed with nucleic acids, polyplexes are formed, mainly posed of cationic polymers such as PEI, PLL, PLO, PAMAM, and PLGA, aiming to achieve loading stability via electrostatic interaction with the genetic material [168]. An example of polyplexes can be observed in the work of Soler Besumbes et al. (2019). In this in vitro study, it was found that cationic PLGA nanoparticles prepared using nanoemulsions (NEs) as a template can be used as delivery systems for DNA vaccines. Thanks to the FDA clearance of its use for medical applications, poly(lactic-co-glycolic acid) (PLGA) is one of the most commonly used synthetic polymers for creating polymeric nanocarriers [169]. Interestingly, macrophages and other antigen-presenting cells readily take up polyplexes, including PLGA nanoparticles (APCs), which are known to generate a more significant and longer-lasting immune response [131]. Another example in the preclinical field is the DNA vaccine against Hepatitis B, which uses the polyplexes of the synthetic polymers poly(-amino ester) (PAE) and poly [2-(dimethylamino) ethyl methacrylate] (PDMAEMA) conjugated with the naturally occurring polysaccharide β-glucan [132]. Although no specific cell-based immune response against the antigen has been developed via subcutaneous vaccination, the bioeffects of vaccine formulations regarding hemocompatibility and cytotoxicity are dose-dependent.

#### 4.2.2. Copolymers

Unlike other formulations, copolymers contain more than one monomer in their composition [170]. They are easy to administer, relatively simple to manufacture, and are known to provide increased adaptive responses [171]. These features can be seen in the study by Hraber et al. (2018), who produced a DNA vaccine containing ZIKV antigens carried by tetrafunctional amphiphilic block copolymers (ABCs). Although ABC has no adjuvant activity [133], this vaccine applied to mice generated a significant increase in uptake by APCs and a consequent elevation in target protein production by the activation of molecular sensors.

#### 4.2.3. Dendrimers

Dendrimers are synthetic macromolecules that are extremely branched, symmetrical, globular, usually cationic, and dendrite-like [134]. They are mainly used as nanocarriers for targeted drug release and the solubilization of poorly water-soluble drugs [164]. Dendrimers are smaller in size than some of the usual nanocarriers, so they can be easily encapsulated to form a nanohybrid and can be surface absorbed or chemically bound [172]. The characteristic three-dimensional structures of dendrimers allow them to pass through cell membranes without generating disruption like conventional polymers [134]. Polymers such as polyamidoamine PAMAM form positively charged dendrimers and easily interact with nucleic acids [173]. However, it has the disadvantage of not being biodegradable and presenting a high molecular weight, which reflects some toxicity besides being recognized by the innate immune system and activating complement [114]. In contrast, Zhao et al. (2020) developed a veterinary DNA vaccine with a poly-L-lysine dendrigraft delivery that enhanced cellular and humoral responses against chickens’ H9N2 avian influenza virus. This generated dendrimer is biodegradable, less toxic, soluble, and non-immunogenic, representing an alternative to commonly used synthetic dendrimers known for their adverse effects.

#### 4.2.4. Polymeric Micelles

Polymeric micelles, made by combining aqueous solution nucleic acids and copolymers (whose properties can be modulated), are often used as mRNA carriers [174]. As such, a novel polymeric micelle based on polyethyleneimine copolymer (PEI) modified with vitamin E succinate (PVES) for mRNA delivery was developed by Ren et al. (2021) for construct evaluation and prophylaxis against SARS-CoV-2. In terms of complement induction and inherent toxicity due to molecular weight and loading, PEI has the same disadvantages as PAMAM [114]. In this construct, however, PEI was modified and, together with vitamin E, which is hydrophobic, formed self-contained amphiphilic copolymers in the micelles, reducing toxicity and inducing T cells, Th1-type immune response, and the amplification of humoral responses against the RBD antigen of SARS-CoV-2.

### 4.3. Microorganism-Based Delivery Systems

Interestingly, although the main activity of delivery systems is to facilitate the specific antigen uptake by immune system cells, some have integrated composition, showing potent immunomodulatory activities [175]. As seen previously, systems that have cationic materials and synthetic polymers such as PEI and PAMAM can be recognized by immune system cells, potentiating the immune response [164]. Besides these, another carrier platform already known since the early days for immunization against infectious agents is currently gaining more visibility for compound delivery. This platform consists of microorganism-based systems, especially those targeting bacteria and yeast [141,176]. Viruses and archaea also present themselves as carrier platforms, mainly in the form of virosomes and archaeosomes, respectively, or through adjunctive carrier proteins [101,177]. Although studies focus more on subunit vaccines, they have been shown to be potentially useful for nucleic acid delivery [177].

As natural adjuvants, they can efficiently activate signaling pathways in immune cells and mobilize the immune system while carrying nucleic acids anchored on the surface or internally [88]. Given their size and complex composition, they are naturally recognized by the defense system and can act as adjuvants by activating pathogen-associated receptors, inducing specific APC uptake [138]. In addition, they amplify the range of administration methods for intranasal, oral, and intravaginal mucosal routes [176]. Some of these organisms are used as immunological adjuvants and food additives and have certification and recognition as safe (GRAS) by the FDA [178,179]. They can be heat-inactivated or modified to ensure host safety to delete their pathogenic components [176,178].

However, although these vectors can induce robust immune responses, some of them, especially viruses and bacteria, can be neutralized by components of the host hu-moral immunity after repeated administration and exhibit some cytotoxicity [80].

#### 4.3.1. Viral Vectors, Virosomes, and Plant Virus Proteins

Viral vector-based technologies are generally considered an effective means of delivering genetic material to cells, but current research uses non-viral systems for nucleic acid vaccine delivery [180]. This preference is because their application is sometimes limited by the development of neutralizing antibodies against the vector, safety concerns, and manufacturing limitations [181]. Although article screening did not retrieve clinical and preclinical trials of viral delivery systems for nucleic acid delivery, virosomes and plant viral nanoparticles have already been employed as carriers [177,182]. Virosomes are nanocarriers that mimic the structure of an enveloped virus whose nucleocapsid has been eliminated [177]. Similarly to liposomes, virosomes are an emerging lipid nanomaterial as an FDA-approved nanocarrier consisting of 60–200 nm unilamellar spherical vesicles [183]. Its preparation techniques are simple, economical, and follow similar major steps [177]. This system interacts with host-cell receptors by transporting nucleic acids, inducing humoral and cellular responses via B cells, and endocytosis by other APCs [184]. On the other hand, plant virus nanoparticles (PVNPs) possess the inherent immunostimulatory capacity and have been investigated as immune adjuvants to stimulate an antitumor immune response [185]. Plant viruses such as CPMV (*Cowpea mosaic virus*) in the form of viral nanoparticles are advantageous for their non-infectivity and lack of toxicity in humans. They have successfully delivered nucleic acid materials by entering cells through alternative pathways or escaping from endosomal vesicles [186].

#### 4.3.2. Archaea, Bacteria, and Their By-Products

As live vectors, bacteria are promising agents for nucleic acid vaccine delivery [176]. The bacterial vector induces a robust immune response due to its natural components, including lipopolysaccharides (LPSs), peptidoglycan, and flagellin, which are recognized by the immune system [187]. Bacteria have specific features called pathogen-associated molecular patterns (PAMPs), recognized by Toll and Nod-like receptors [188]. This introduction induces the native immune response and enhances the adaptive immune response [136]. However, for some species, the precise mechanisms by which bacterial vectors make nucleic acids available in host cells are not yet fully understood [176]. Notably, recombinant and attenuated strains, such as some species of *Salmonella*, *Mycobacterium*, *Yersinia, Listeria*, and *Shigella*, and non-pathogenic bacteria, such as lactic acid bacteria (LAB), are considered carriers of nucleic acid vaccines [136,176].

These organisms also benefit the administration route via the mucosal route that beneficially induces mucosal and systemic immune responses [176]. LABs, for example, are even more suitable because, in addition to being non-pathogenic, withstanding acidic conditions in the gastrointestinal (GI) system, and protecting nucleic acids, they also exhibit probiotic effects [189]. In addition to these, bacterial derivatives can be used as potentially useful nanocarriers for antigen delivery, some as “bacterial ghosts” that possess the ability to stimulate immune responses as potent as those of live bacteria [190]. Other components such as S-layer, endospores, and outer membrane vesicles (OMVs) are also applied, but the latter contains LPS which can cause immune toxicity [188].

In the field of vaccination, OMVs were recently used in the study by Li et al. (2022) to deliver mRNA vaccine and lysosomal escape protein. With this construct, melanoma progression was significantly inhibited and caused regression in a mouse model of colon cancer. As a DNA vaccine carrier, attenuated *Salmonella typhimuriumaro* A was used in the prophylactic vaccine against Duck Tembusu virus (DTMUV) and administered orally, inducing strong humoral responses with a high level of specific antibodies against the antigen [136]. This Gram-negative bacterium demonstrates strong adjuvant activities and contains LPS which is recognized by the TLR4 receptor, and flagellin, which binds to TLR5. These bindings activate pathways that induce cytokine release and potentiate the immune response [176]. Already in prophylaxis, a DNA vaccine carried and expressed by *Mycobacterium paragordonae* administered subcutaneously against SARS-CoV-2 generated a robust cellular response and Th1 induction in mice [137].

However, while bacterial delivery systems have several advantages, such as potent immune response induction, oral delivery, and the increased targeting of APCs, they also have significant disadvantages that must be considered [187]. First, the use of live bacteria, albeit in modified or attenuated form, includes the likelihood of causing infection, particularly in infants and immunocompromised patients [176]. Second, bacterial delivery systems, such as viruses, develop neutralizing antibodies against the bacteria (vector) itself, resulting in the decreased efficacy of the vaccine antigen [80].

Archaea, a domain of single-celled living things morphologically similar to bacteria but genetically distinct, are also used as vaccine delivery systems, especially in the form of archaeosomes [191]. The archaeosome is a liposome-based nano-delivery system developed for gene delivery. For example, Karimi et al. (2020) produced a therapeutic DNA vaccine carried on the surface or encapsulated by an Archeosome against HPV. This vaccine, administered subcutaneously, induced a strong humoral, cellular, and Th1-oriented polarization response.

#### 4.3.3. Yeasts

Yeasts are versatile single-celled microorganisms commonly used in the food industry that have potential application value as biofactories of therapeutic proteins and carriers of biological molecules [179]. Some strains have been certified as generally recognized as safe (GRAS) by the FDA and have been used for bioproduction [192]. *Yarrowia lipolytica*, *Schizosaccharomyces pombe*, *Kluyveromyces lactis*, *Pichia pastoris*, and *Saccharomyces cerevisiae* are predominantly used as carriers for drug delivery, subunit vaccine production, and recombinant proteins [178,192]. In addition, they have been evaluated as a vehicle for nucleic acids, presenting several advantages, such as oral delivery capability, adjuvant activity, absence of toxicity, and specific delivery [178].

Yeast-based delivery vehicles are excellent candidates for oral vaccines as they possess cellular characteristics that confer greater resistance to enzymatic digestion, acidic environments, and mucosal barriers of the gastrointestinal tract [139]. Their adjuvant activity is obtained by the various components that can stimulate or modulate the host immune response [193]. The cell wall mainly comprises β-1,3 and β-1,6-glucan, chitin, mannan, and other polysaccharides [179]. These promote immunostimulation by binding with dectin receptors, mannose-fucose, and Toll-like receptors (TLRs) such as TLR-2, 4, and 6 in DCs [138]. This interaction leads to the secretion of Th1 and Th17-type cytokines through pathogen-associated molecular patterns (PAMPs) and the activation of innate immunity components, inducing a specific APC uptake [192]. After being phagocytosed, they are degraded by phagolysosomes, and the nucleic acid is released and transported to their synthesis regions [178].

* S. cerevisiae* is the most widely used non-pathogenic yeast species for the delivery of nucleic acid vaccines, either in whole-cell recombinant form or in capsule, microcapsule, or surface display system configuration [194]. Currently, the studies of DNA and RNA vaccines delivered by this strain encompass both prophylaxis and therapy against the diseases of humans and other animals, such as fish [140]. For example, Han et al. (2019), for example, developed an oral DNA vaccine based on whole *S. cerevisiae* that induced antigen-specific responses, protecting carp against *Aeromonas hydrophila* infection. In the field of post-transcriptional control therapy, a vaccine of siRNA and Trp2 coated with PEI nanoparticles and indoleamine 2,3-dioxygenase (IDO) delivered by recombinant *S. cerevisiae* inhibited IDO expression and induced T-cell responses against melanoma in mice [138]. In turn, Zhang et al. (2021) produced a vaccine composed of IL-1β shRNA delivered by *S. cerevisiae* microcapsules for post-traumatic osteoarthritis therapy and verified the regulation of the inflammatory response and IL-1β expression by the oral route. Finally, a therapeutic DNA vaccine in the preclinical phase was carried out to regulate myostatin and generate immune modulation by suppressing IL-21 [140]. As for safety, these vaccines did not produce adverse effects, and because they are orally or subcutaneously delivered, they decreased discomfort, making application easier.

However, although yeast release transporters show great potential, there is still a need to research and understand their observed advantages, as well as a better evaluation of the amount of genetic material to be carried, specific uptake in the body, and their transport in vivo [179].

## 5. The Influence of Delivery Systems on the Immune Response

**Figure 2:**
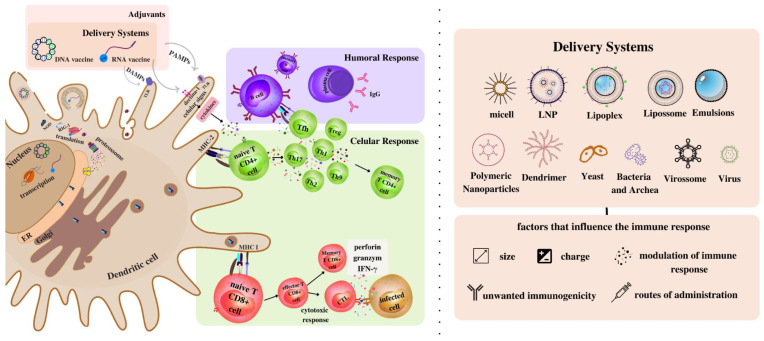
Activation of immune pathways generated by nucleic acid vaccines with delivery systems. After vaccine administration, nucleic acids are introduced into the dendritic cell or other APCs via delivery systems that can influence the immune response depending on their characteristics such as charge, size, and molecular patterns. In a DNA vaccine, the delivery systems should facilitate the entry of the vaccine into the cell and favor the access of the genetic material to the nucleus to be transcribed into mRNA. Then, the routes for DNA and mRNA vaccines are the same, with the translation of the antigen protein occurring in the cytoplasm, processing by proteasomes, and the presentation of the epitopes by MCH-I. This presentation activates naive CD8+ T cells, leading to the production of effector cells, the induction of cytotoxic responses, and the expansion of memory CD8+ T cells. On the other hand, exogenous proteins released by transfected cells can be directly recognized by B cells or phagocytized by DCs and processed and presented by MHC-II. In this case, they can activate antigen-specific CD4+ T cells that expand into corresponding subtypes, release cytokines, and interact with B cells, leading to a strong humoral response.

To achieve efficient delivery, the development of a platform that overcomes existing barriers is of great importance [115]. Furthermore, these systems must allow for the transport of nucleic acids to their protein synthesis sites. For the DNA vaccine, it is necessary to overcome the cell membrane and then the nuclear membrane for the transcription of DNA into mRNA to occur, which will contain the information in codons for the synthesis of the antigen (Figure 2) [53]. Differently, mRNA vaccine systems must provide greater stability to this macromolecule, ensuring its passage through the cell membrane for its translation to occur in the cytoplasm [74].

Some systems, such as liposomes, LNPs, and lipoplexes, have amphiphilic characteristics and can penetrate the cell through the cell membrane. Others, such as those based on microorganisms, are identified and phagocytized by APCs [51]. Taking into consideration the size and recognition of APCs by pathogens, particles of 20–200 nm, similar to viral sizes, are usually ingested by dendritic cells (DCs), while macrophages usually engulf larger particles (0.5–5 µm) [195]. The size of the vaccine particles also determines the route of antigen transport to the lymph nodes. Smaller particles generally enter circulation through the peripheral capillaries, while those sizes 10–200 nm travel to the lymphatic capillaries [196]. Because there are so many APCs and T and B cells in these capillaries, particles that reach the lymph nodes elicit stronger responses [197].

Surface charge, another vital property of vaccine delivery systems, also exhibits adverse effects on the targeting process of antigen uptake by APCs [198]. The surface charge has been proven to be one of the main factors influencing its internalization efficiency by APCs and cytotoxicity [199]. Cationic particles, such as those present in lipid- and polymer-based systems, are better internalized by APCs but are also more prone to induce aggregation and hemolysis [200,201]. Mechanistically, the high cytotoxicity of positively charged materials is mainly correlated with the disruption of the negative charge of cell membranes during the penetration process, which induces cell death [202]. Negatively charged materials are more favorable for better interstitial movement for improved lymphatic uptake delivery performance and retention in lymph nodes, which harbor cells relevant to infection control [203]. However, because nucleic acids have a net negative charge, their stability is best ensured by electrostatic interaction with cationic materials [204]. Major safety concerns associated with the various delivery systems are biocompatibility, biodistribution, and the induction of unwanted immune reactions [51]. For example, the repeated application of adenoviral, bacterial, and PLL- and PAMAM-based systems can neutralize the carrier molecule and not the target antigen [80,114]. In therapeutic vaccines that require larger applications at shorter intervals, this unrequited neutralization may interfere with the immune response. On the other hand, in prophylactic vaccines, vaccine applications generally have longer gaps between doses, and therefore, may be less impacted.

Physical delivery methods such as gene gun and electroporation can facilitate the delivery of the genetic material to APCs and enhance the immune response [48]. Physical delivery methods such as gene gun and electroporation can facilitate the delivery of the genetic material to APCs and enhance the immune response [48]. Trimble et al. (2015) conducted a study to see whether VGX-3100, synthetic plasmids targeting HPV-16 and HPV-18 E6 and E7 proteins delivered via electroporation, would cause histopathological regression in women with CINs 2 and 3. The results showed immune responses in peripheral blood (both CD8+ T cells and antibodies) and cervical tissue that correlated with histopathological regression and viral clearance [205].

Regarding the gene gun method, Lee et al. (2013) showed that HPV-16 E7-expressing murine TC-1 tumor-bearing mice were orally treated with AR-42 and/or CRT/E7 DNA vaccines via gene gun. This study demonstrated that the treatment with AR-42 and CRT/E7 DNA vaccines combined reduced tumor growth and improved survival in mice [206]. Furthermore, the combination treatment generated increased E7-specific CD8+ T cells and increased T-cell-mediated cytotoxicity. In another example, demonstrated by Garza-Morales et al. (2019), mice were immunized with the SP-SA-E7-4-1BBL DNA construct using the gene gun delivery system. In this study, robust therapeutic and prophylactic effects were observed against TC-1 tumors expressing HPV-16 E7 when compared to controls containing E7wt or SP-SA-4-1BBL alone [207].

However, these delivery methods require expensive and specific equipment that may not be practical for large-scale vaccination campaigns [124]. Vaccines carried by the delivery systems mentioned here are primarily administered to patients in two main routes of administration, intramuscular and subcutaneous, followed by oral and intranasal vaccinations. To transfect as many APCs as possible, the delivery of nucleic acid vaccines to secondary lymphoid organs via systemic application such as intravenous, oral, or pulmonary administration may be an appropriate strategy [118]. Oral vaccines are valued in developing countries as potential strategies for improving the effectiveness of vaccine delivery, reducing administration costs, and increasing vaccine adherence [208]. However, the oral route may not be the most suitable route for targeting lymph nodes given the existence of gastrointestinal tract barriers, low pH, and the presence of enzymes [192]. Systems based on bacteria can administer nucleic acid vaccines orally; however, they are more susceptible to chemical and physical barriers than yeast-containing β-glucan [176]. Because β-glucan resists acids more strongly and is not digested in the stomach, the phagocytosis process by M cells is facilitated, leading to vaccine entry into the lymphatic system [179].

Finally, some delivery systems, in addition to acting as carrier molecules, have inherent adjuvant characteristics and can therefore assist in polarizing the immune response [175]. Depending on the composition of the carrier molecule, they can be recognized by DCs by receptors such as Toll-like receptors (TLRs), NOD, RIG, and Dectin-1 by identifying molecular patterns (Figure 2). In this case, the signals received by the receptors induce the release of cytokines, which interact with the cells of the adaptive system, altering the balance and inducing the immune response. In this review, most responses elicited by DNA and RNA vaccines with the various delivery systems were toward Th1 or more balanced Th2/Th1 responses. Delivery systems that assist in the induction of Th1 responses are mainly targeted for fighting cancer cells and against some intracellular pathogens such as viruses by influencing cellular and cytotoxic responses [111]. For example, in SARS-CoV-2, this response is notably more interesting, given that vaccines with Th2 bias adjuvants have shown an association with enhanced lung pathogenesis [124]. However, other vaccines require the stronger activation and maintenance of humoral immunity, necessary to clear extracellular infections such as parasites and allergens, and so, in this case, a Th2 response or a more balanced bias may be required [30].

## 6. Conclusions

Unlike preventive vaccinations, therapeutic vaccines attempt to trigger a biological response against altered cells. The use of therapeutic vaccines combined with delivery methods facilitates the activation and recruitment of T cells against antigens, thus eliciting a more direct antigen-specific response. In this context, a better knowledge of immunological pathways in creating innovative and alternative therapeutics is vital to enhance disease management and survival in patients with HPV-related malignancies. The molecular organization of delivery systems determines their interaction with nucleic acids, their transport to areas of antigen formation, how they are recognized by immune cells, and the creation of inflammation, and cytotoxic consequences. Among the known transporters, lipid-based transporters are the most extensively employed for delivering nucleic acid vaccines. However, the cargo-dependent toxicity of these particles creates cytotoxic effects and generates instability, requiring alterations that may affect their efficacy.

Furthermore, the interaction of components of the delivery systems with the cells of the adaptive immune system might impact the balance and activation of the immune response. Thus, using microorganisms with natural adjuvant action and GRAS status, such as yeast, since they have fewer cytotoxic effects than other delivery platforms, may be delivered orally and elicit robust adaptive responses. Therefore, in addition to knowing the immune response generated by the pathogen, it is vital to evaluate the type of delivery method to be employed, its cost-effectiveness, its impact on the immune response, and the intended result for the vaccine designs to generate optimal immune responses.

## Figures and Tables

**Table 1:** Examples of Clinical Trials of Therapeutic DNA/RNA Vaccines against HPV-Associated Malignancies.

Nucleic Acid	Antigen and Delivery	Immune Response	Population	Main Results	Clinical Phase	Ref.
DNA	MEDI0457 HPV-16/18 E6 and E7 pDNA co-injected IL-12 pDNA followed by electroporation with the CELLECTRA device	Increased humoral and specific T cell responses	Cervical cancer patients after chemoradiation	The vaccine was well tolerated; the concurrent treatment with chemoradiation was associated with specific and increased adaptive responses	I (NCT02172911)	[63]
		Induction of HPV-specific CD8+ and PD-1+ T cells	Patients with p16 + locally advanced HNSCC	Immunization generated mild reactions at the injection site but no adverse events; promoted the induction of durable and antigen-specific peripheral and tumor immune responses	I/IIa (NCT02163057)	[64]
	DNA vaccine with two plasmid constructs containing HPV16 E6 and E7 with sig and KDEL and three CD4 helper sequences, administered by DNA tattoo vaccine.	In five of six patients who responded clinically, E6/E7-specific CD4+ and CD8+ T cell reactivity could be detected	Patients with usual vulvar intraepithelial neoplasia (uVIN) HPV16+	Vaccines were well tolerated; clinical responses were observed in 43% of patients, with 2 complete responses and 4 partial responses; 35.71% of patients had HPV-specific T cell responses in their blood	I/II (NTR 4607)	[65]
	GX-188E (Genexine, Inc.) HPV type 16/18 E6/E7 DNA therapeutic vaccine	Patients with histopathological regression showed statistically significant increases in HPV-specific IFNγ responses, but these were not correlated with histological regression of cervical lesions	CIN3 positive patients for HPV 16/18	Among patients, 52% at 20 weeks after the first injection and 67% at 36 weeks after the first injection showed histopathological regression after receiving the vaccine	II (NCT02139267)	[61]
	GX-188E combination (Genexine, Inc.) intramuscularly + pembrolizumab	Induced human papillomavirus (HPV) E6- and E7-specific T cell responses	HPV-16+ or HPV-18+ patients with inoperable, metastatic advanced cervical cancer	The combination vaccine was safe and treatment-related adverse events were manageable, showing preliminary antitumor activity	Ib-II (NCT03444376)	[66]
RNA	BNT113 RNA vaccine Anti-CD40 against HPV	Results not yet expected	HPV16+ HNSCCs patients, and with head and neck cancer, cervical, anogenital, and recurrent penile cancer	Results not yet expected	I recruiting (NCT03418480)	[67]
	Vvax001 replicon RNA vaccine based on Semliki Forest virus (SFV) encoding HPV16-derived E6 and E7 antigens)	It resulted in CD4+ and CD8+ T cell responses to specific antigens; even small doses of the infectious particles elicited E6/E7-specific interferon (IFN)-γ responses	Participants with a history of cervical intraepithelial neoplasia	Immunization was safe and well tolerated, resulting in positive vaccine-induced immune responses in all patients	I (NCT03141463)	[68]

**Table 2:** Recent studies of nucleic acid vaccines with lipid, polymer, and microorganism-based delivery systems.

Delivery Systems		Vaccine Type	Immune Response	Charge	Size	Routes	Phase	Ref.
**Lipid-Based**	**Liposomes**	RNA—Lipo-MERIT- FixVac (BNT111) against malignant melanoma	Cellular response	-	200–400 nm	i.v.	c. F-I	[120]
	**Hybrids of Liposomes**	DNA—ssPalmE-LNP TgGRA15 against *Toxoplasma gondii*	Humoral and Th1-type	+	140 nm	s.c.	p.c	[121]
		DNA with DDA-MPLA-TDB against tuberculosis	Th1 and memory T-cell production	+	417 ± 60 nm	i.m.	p.c	[122]
		DNA with hybrid liposome–polymer nanoparticle (pSFV-MEG/LNPs) against traveler’s diarrhea	Humoral and cellular response	-	161.61 ± 15.63 nm	i.m.	p.c	[123]
	**Lipoplexes**	DNA against SARS-CoV-2 with DOTAP-based cationic liposomes	Humoral and cellular	+	130.9 ± 5.8 nm	i.m.	p.c	[124]
			Th1/Th2 response in balance					
		Lipopolyplexes with RNA Vaccine encoding influenza antigen	Specific T-cell induction	neutral	180/10 nm	i.m.	p.c	[125]
	**Emulsions**	DNA against HIV coadministered with protein + MF59	No direct relation to the immune response	n.i	±160 nm	i.m.	c. F-I	[126]
	**Cationic Nanoemulsions**	DNA therapy against mucopolysaccharidosis type I	Increased enzyme expression	+	150 ± 265 nm	i.n.	p.c	[127]
	**Lipid Nanoparticles**	mRNA-1273 prophylactic vaccine against COVID-19	Humoral and cellular and Th1 type	+	59 ± 100 nm	i.m.	c. F-III ap	[128]
		BNT162 (3 LNP–mRNA)	Humoral and cellular and Th1 type	+	60 ± 100 nm	i.m.	c. F-III ap	[129]
		DNA for immunoprophylaxis in animals using LUNAR ®	Increased cellular response	n.i	n.i	i.m.	p.c	[130]
**Polymer Based**	**Polyplexes**	DNA therapeutics with cationic polymeric NPs against Leishmaniasis	*	+	125 ± 130 nm	*	i.vt	[131]
		DNA against hepatitis B using PDMAEMA:PβAE polyplexes with β-glucan	No specific cell-based immune response against the antigen	+	≈180 nm	s.c	p.c	[132]
	**Copolymer**	Tetrafunctional ABC DNA against Zika virus	Increased humoral response	-	50 nm	i.m.	p.c	[133]
	**Dendrimer**	Dendrigraft DNA against avian influenza virus H9N2 in chickens	Humoral and strong cellular immune response	+	68.9 ± 2.1 nm	i.m.	p.c	[134]
	**Polymeric Micelles**	mRNA with polyethyleneimine modified with vitamin E succinate (PVES) against SARS-CoV-2	Th1 cellular response and humoral responses	+	144.7 ± 0.76 nm	i.m.	p.c	[135]
**Microorganism-Based**	**Bacteria**	Prophylactic DNA delivered by *Salmonella typhimuriumaro* A attenuated against Duck Tembusuvirus (DTMUV)	Humoral with high level of specific antibodies	*	2–5 μm	o	p.c	[136]
		DNA carried and expressed by *Mycobacterium paragordonae* against SARS-CoV-2	Strong cellular response and Th1 induction	*	2–3 μm	s.c	p.c	[137]
	**Archeas**	Archeosome-encapsulated or surface-loaded therapeutic DNA against HPV	Strong humoral and cellular response, induction of Th1	-	(S) 127 ± 2.1 nm e	s.c	p.c	[101]
					(E) 429 ± 5 nm			
	**Yeasts**	Therapeutic vaccine of siRNA and Trp2 coated with (PEI)-IDO and delivered by recombinant *S. cerevisiae*	Inhibited IDO expression, decreased Treg generation and induced T cell responses	+	140 ± 20.0 nm	s.c	p.c	[138]
		Veterinary prophylactic DNA delivered by *S. cerevisiae* against *A. hydrophila*	Induced antigen-specific humoral responses	-	2–5 μm	o	p.c	[139]
		DNA therapeutics carried by *S. cerevisiae* to regulate myostatin	Inhibition of myostatin protein and modulation by IL21 suppression	-	2–5 μm	o	p.c	[140]
		IL-1β shRNA vaccine delivered by *S. cerevisiae* microcapsules for therapy of post-traumatic osteoarthritis	Regulated the inflammatory response and reduced IL-1β expression	-	n.i	o	p.c	[141]

* i.v. (intravascular), i.m. (intramuscular), o (oral), i.n. (intranasal), s.c. (subcutaneous), n.i (not informed), c. F-I (Clinic Phase I), c.F-III ap (Clinic phase III, application), p.c (preclinical).

## Data Availability

Not applicable.

## References

[1] Sung H., Ferlay J., Siegel R.L., Laversanne M., Soerjomataram I., Jemal A., Bray F. (2021). Global Cancer Statistics 2020: GLOBOCAN Estimates of Incidence and Mortality Worldwide for 36 Cancers in 185 Countries. CA Cancer J. Clin..

[2] de Martel C., Georges D., Bray F., Ferlay J., Clifford G.M. (2020). Global Burden of Cancer Attributable to Infections in 2018: A Worldwide Incidence Analysis. Lancet Glob. Health.

[3] Araldi R.P., Sant’Ana T.A., Módolo D.G., de Melo T.C., Spadacci-Morena D.D., de Cassia Stocco R., Cerutti J.M., de Souza E.B. (2018). The Human Papillomavirus (HPV)-Related Cancer Biology: An Overview. Biomed. Pharmacother..

[4] de Villiers E.-M., Fauquet C., Broker T.R., Bernard H.-U., zur Hausen H. (2004). Classification of Papillomaviruses. Virology.

[5] Bernard H.-U., Burk R.D., Chen Z., van Doorslaer K., Hausen H.Z., de Villiers E.-M. (2010). Classification of Papillomaviruses (PVs) Based on 189 PV Types and Proposal of Taxonomic Amendments. Virology.

[6] Münger K., Baldwin A., Edwards K.M., Hayakawa H., Nguyen C.L., Owens M., Grace M., Huh K. (2004). Mechanisms of Human Papillomavirus-Induced Oncogenesis. J. Virol..

[7] Li N., Franceschi S., Howell-Jones R., Snijders P.J.F., Clifford G.M. (2011). Human Papillomavirus Type Distribution in 30,848 Invasive Cervical Cancers Worldwide: Variation by Geographical Region, Histological Type and Year of Publication. Int. J. Cancer.

[8] Clifford G., Gallus S., Herrero R., Muñoz N., Snijders P., Vaccarella S., Anh P., Ferreccio C., Hieu N., Matos E. (2005). Worldwide Distribution of Human Papillomavirus Types in Cytologically Normal Women in the International Agency for Research on Cancer HPV Prevalence Surveys: A Pooled Analysis. Lancet.

[9] Hirth J. (2019). Disparities in HPV Vaccination Rates and HPV Prevalence in the United States: A Review of the Literature. Hum. Vaccines Immunother..

[10] Shamseddine A.A., Burman B., Lee N.Y., Zamarin D., Riaz N. (2021). Tumor Immunity and Immunotherapy for HPV-Related Cancers. Cancer Discov..

[11] Chesson H.W., Dunne E.F., Hariri S., Markowitz L.E. (2014). The Estimated Lifetime Probability of Acquiring Human Papillomavirus in the United States. Sex. Transm. Dis..

[12] Best S.R., Niparko K.J., Pai S.I. (2012). Biology of Human Papillomavirus Infection and Immune Therapy for HPV-Related Head and Neck Cancers. Otolaryngol. Clin. N. Am..

[13] Ghebre R., Berry-Lawhorn J.M., D’Souza G. (2021). State of the Science: Screening, Surveillance, and Epidemiology of HPV-Related Malignancies. Am. Soc. Clin. Oncol. Educ. Book.

[14] Chen M.-L., Wang S.-H., Wei J.C.-C., Yip H.-T., Hung Y.-M., Chang R. (2021). The Impact of Human Papillomavirus Infection on Skin Cancer: A Population-Based Cohort Study. Oncologist.

[15] de Oliveira T.H.A., do Amaral C.M., de França São Marcos B., Nascimento K.C.G., de Miranda Rios A.C., Quixabeira D.C.A., Muniz M.T.C., da Silva Neto J.C., de Freitas A.C. (2018). Presence and Activity of HPV in Primary Lung Cancer. J. Cancer Res. Clin. Oncol..

[16] Guo L., Liu S., Zhang S., Chen Q., Zhang M., Quan P., Sun X.-B. (2016). Human Papillomavirus-Related Esophageal Cancer Survival: A Systematic Review and Meta-Analysis. Medicine.

[17] Lei J., Ploner A., Elfström K.M., Wang J., Roth A., Fang F., Sundström K., Dillner J., Sparén P. (2020). HPV Vaccination and the Risk of Invasive Cervical Cancer. N. Engl. J. Med..

[18] Cheng L., Wang Y., Du J. (2020). Human Papillomavirus Vaccines: An Updated Review. Vaccines.

[19] Markowitz L.E., Schiller J.T. (2021). Human Papillomavirus Vaccines. J. Infect. Dis..

[20] Ornellas P., Ornellas A.A. (2018). HPV Vaccination Is Fundamental for Reducing or Erradicate Penile Cancer | Opinion: NO. Int. Braz. J. Urol..

[21] Bewley S. (2022). HPV Vaccination and Cervical Cancer Screening. Lancet.

[22] Catarino R., Petignat P., Dongui G., Vassilakos P. (2015). Cervical Cancer Screening in Developing Countries at a Crossroad: Emerging Technologies and Policy Choices. World J. Clin. Oncol..

[23] Boilesen D.R., Nielsen K.N., Holst P.J. (2021). Novel Antigenic Targets of HPV Therapeutic Vaccines. Vaccines.

[24] Khairkhah N., Bolhassani A., Najafipour R. (2022). Current and Future Direction in Treatment of HPV-Related Cervical Disease. J. Mol. Med..

[25] Lee J., Kumar S.A., Jhan Y.Y., Bishop C.J. (2018). Engineering DNA Vaccines against Infectious Diseases. Acta Biomater..

[26] Vetter V., Denizer G., Friedland L.R., Krishnan J., Shapiro M. (2018). Understanding Modern-Day Vaccines: What You Need to Know. Ann. Med..

[27] Li L., Petrovsky N. (2017). Molecular Adjuvants for DNA Vaccines. Curr. Issues Mol. Biol..

[28] Schijns V., Fernández-Tejada A., Barjaktarović Ž., Bouzalas I., Brimnes J., Chernysh S., Gizurarson S., Gursel I., Jakopin Ž., Lawrenz M. (2020). Modulation of Immune Responses Using Adjuvants to Facilitate Therapeutic Vaccination. Immunol. Rev..

[29] Rosales C., Rosales R., Afrin F., Hemeg H., Ozbak H. (2017). Prophylactic and Therapeutic Vaccines against Human Papillomavirus Infections. Vaccines.

[30] Tanner R., Villarreal-Ramos B., Vordermeier H.M., McShane H. (2019). The Humoral Immune Response to BCG Vaccination. Front. Immunol..

[31] Pulendran B., Arunachalam P.S., O’Hagan D.T. (2021). Emerging Concepts in the Science of Vaccine Adjuvants. Nat. Rev. Drug Discov..

[32] Wang J.W., Roden R.B. (2013). Virus-like Particles for the Prevention of Human Papillomavirus-Associated Malignancies. Expert Rev. Vaccines.

[33] Buck C.B., Day P.M., Trus B.L. (2013). The Papillomavirus Major Capsid Protein L1. Virology.

[34] Hladíková K., Koucký V., Bouček J., Laco J., Grega M., Hodek M., Zábrodský M., Vošmik M., Rozkošová K., Vošmiková H. (2019). Tumor-Infiltrating B Cells Affect the Progression of Oropharyngeal Squamous Cell Carcinoma via Cell-to-Cell Interactions with CD8+ T Cells. J. Immunother. Cancer.

[35] Kim S.S., Shen S., Miyauchi S., Sanders P.D., Franiak-Pietryga I., Mell L., Gutkind J.S., Cohen E.E.W., Califano J.A., Sharabi A.B. (2020). B Cells Improve Overall Survival in HPV-Associated Squamous Cell Carcinomas and Are Activated by Radiation and PD-1 Blockade. Clin. Cancer Res..

[36] Kamphorst A.O., Araki K., Ahmed R. (2015). Beyond Adjuvants: Immunomodulation Strategies to Enhance T Cell Immunity. Vaccine.

[37] Boukhebza H., Bellon N., Limacher J.M., Inchauspé G. (2012). Therapeutic Vaccination to Treat Chronic Infectious Diseases: Current Clinical Developments Using MVA-Based Vaccines. Hum. Vaccines Immunother..

[38] Eggenhuizen P.J., Ng B.H., Ooi J.D. (2020). Treg Enhancing Therapies to Treat Autoimmune Diseases. Int. J. Mol. Sci..

[39] Hu Z., Ott P.A., Wu C.J. (2018). Towards Personalized, Tumour-Specific, Therapeutic Vaccines for Cancer. Nat. Rev. Immunol..

[40] Yim E.-K., Park J.-S. (2005). The Role of HPV E6 and E7 Oncoproteins in HPV-Associated Cervical Carcinogenesis. Cancer Res. Treat..

[41] Yeo-Teh N., Ito Y., Jha S. (2018). High-Risk Human Papillomaviral Oncogenes E6 and E7 Target Key Cellular Pathways to Achieve Oncogenesis. Int. J. Mol. Sci..

[42] Paolini F., Curzio G., Cordeiro M.N., Massa S., Mariani L., Pimpinelli F., de Freitas A.C., Franconi R., Venuti A. (2017). HPV 16 E5 Oncoprotein Is Expressed in Early Stage Carcinogenesis and Can Be a Target of Immunotherapy. Hum. Vaccines Immunother..

[43] Rumfield C.S., Roller N., Pellom S.T., Schlom J., Jochems C. (2020). Therapeutic Vaccines for HPV-Associated Malignancies. ImmunoTargets Ther..

[44] Donà M.G., Di Bonito P., Chiantore M.V., Amici C., Accardi L. (2021). Targeting Human Papillomavirus-Associated Cancer by Oncoprotein-Specific Recombinant Antibodies. Int. J. Mol. Sci..

[45] Melssen M., Slingluff C.L. (2017). Vaccines Targeting Helper T Cells for Cancer Immunotherapy. Curr. Opin. Immunol..

[46] Brinkman J.A., Hughes S.H., Stone P., Caffrey A.S., Muderspach L.I., Roman L.D., Weber J.S., Kast W.M. (2007). Therapeutic Vaccination for HPV Induced Cervical Cancers. Dis. Markers.

[47] Pan C., Yue H., Zhu L., Ma G., Wang H. (2021). Prophylactic Vaccine Delivery Systems against Epidemic Infectious Diseases. Adv. Drug Deliv. Rev..

[48] Qin F., Xia F., Chen H., Cui B., Feng Y., Zhang P., Chen J., Luo M. (2021). A Guide to Nucleic Acid Vaccines in the Prevention and Treatment of Infectious Diseases and Cancers: From Basic Principles to Current Applications. Front. Cell Dev. Biol..

[49] Zhang N., Nandakumar K.S. (2018). Recent Advances in the Development of Vaccines for Chronic Inflammatory Autoimmune Diseases. Vaccine.

[50] Zhang C., Maruggi G., Shan H., Li J. (2019). Advances in MRNA Vaccines for Infectious Diseases. Front. Immunol..

[51] Hobernik D., Bros M. (2018). DNA Vaccines—How Far From Clinical Use?. Int. J. Mol. Sci..

[52] Bai H., Lester G.M.S., Petishnok L.C., Dean D.A. (2017). Cytoplasmic Transport and Nuclear Import of Plasmid DNA. Biosci. Rep..

[53] Ghaffarifar F. (2018). Plasmid DNA Vaccines: Where Are We Now?. Drugs Today.

[54] Lopes A., Vandermeulen G., Préat V. (2019). Cancer DNA Vaccines: Current Preclinical and Clinical Developments and Future Perspectives. J. Exp. Clin. Cancer Res..

[55] Faurez F., Dory D., Le Moigne V., Gravier R., Jestin A. (2010). Biosafety of DNA Vaccines: New Generation of DNA Vectors and Current Knowledge on the Fate of Plasmids after Injection. Vaccine.

[56] Shafaati M., Saidijam M., Soleimani M., Hazrati F., Mirzaei R., Amirheidari B., Tanzadehpanah H., Karampoor S., Kazemi S., Yavari B. (2022). A Brief Review on DNA Vaccines in the Era of COVID-19. Future Virol..

[57] Wang Z., Troilo P.J., Wang X., Griffiths T.G., Pacchione S.J., Barnum A.B., Harper L.B., Pauley C.J., Niu Z., Denisova L. (2004). Detection of Integration of Plasmid DNA into Host Genomic DNA Following Intramuscular Injection and Electroporation. Gene Ther..

[58] Khobragade A., Bhate S., Ramaiah V., Deshpande S., Giri K., Phophle H., Supe P., Godara I., Revanna R., Nagarkar R. (2022). Efficacy, Safety, and Immunogenicity of the DNA SARS-CoV-2 Vaccine (ZyCoV-D): The Interim Efficacy Results of a Phase 3, Randomised, Double-Blind, Placebo-Controlled Study in India. Lancet.

[59] Aida V., Pliasas V.C., Neasham P.J., North J.F., McWhorter K.L., Glover S.R., Kyriakis C.S. (2021). Novel Vaccine Technologies in Veterinary Medicine: A Herald to Human Medicine Vaccines. Front. Vet. Sci..

[60] Porter K.R., Raviprakash K. (2017). DNA Vaccine Delivery and Improved Immunogenicity. Curr. Issues Mol. Biol..

[61] Choi Y.J., Hur S.Y., Kim T.-J., Hong S.R., Lee J.K., Cho C.-H., Park K.S., Woo J.W., Sung Y.C., Suh Y.S. (2020). A Phase II, Prospective, Randomized, Multicenter, Open-Label Study of GX-188E, an HPV DNA Vaccine, in Patients with Cervical Intraepithelial Neoplasia 3. Clin. Cancer Res..

[62] INOVIO Pharmaceuticals, Inc. (2021). INOVIO Announces Positive Results from REVEAL 1, A Phase 3 Pivotal Trial Evaluating VGX-3100, Its DNAbased HPV Immunotherapy for the Treatment of High-Grade Precancerous Cervical Dysplasia Caused by HPV-16 and/or HPV-18.

[63] Hasan Y., Furtado L., Tergas A., Lee N., Brooks R., McCall A., Golden D., Jolly S., Fleming G., Morrow M. (2020). A Phase 1 Trial Assessing the Safety and Tolerability of a Therapeutic DNA Vaccination against HPV16 and HPV18 E6/E7 Oncogenes after Chemoradiation for Cervical Cancer. Int. J. Radiat. Oncol. Biol. Phys..

[64] Aggarwal C., Cohen R.B., Morrow M.P., Kraynyak K.A., Sylvester A.J., Knoblock D.M., Bauml J.M., Weinstein G.S., Lin A., Boyer J. (2019). Immunotherapy Targeting HPV16/18 Generates Potent Immune Responses in HPV-Associated Head and Neck Cancer. Clin. Cancer Res..

[65] Bakker N.A.M., Rotman J., van Beurden M., Zijlmans H.J.M., van Ruiten M., Samuels S., Nuijen B., Beijnen J., De Visser K., Haanen J. (2021). HPV-16 E6/E7 DNA Tattoo Vaccination Using Genetically Optimized Vaccines Elicit Clinical and Immunological Responses in Patients with Usual Vulvar Intraepithelial Neoplasia (UVIN): A Phase I/II Clinical Trial. J. Immunother. Cancer.

[66] Youn J.W., Hur S.-Y., Woo J.W., Kim Y.-M., Lim M.C., Park S.Y., Seo S.S., No J.H., Kim B.-G., Lee J.-K. (2020). Pembrolizumab plus GX-188E Therapeutic DNA Vaccine in Patients with HPV-16-Positive or HPV-18-Positive Advanced Cervical Cancer: Interim Results of a Single-Arm, Phase 2 Trial. Lancet Oncol..

[67] Lee K.-A. Therapeutic HPV Vaccine Trial ± Anti-CD40 in HPV-Driven Squamous Cell Carcinoma. 2016, Unpublished work.

[68] Komdeur F.L., Singh A., van de Wall S., Meulenberg J.J.M., Boerma A., Hoogeboom B.N., Paijens S.T., Oyarce C., de Bruyn M., Schuuring E. (2021). First-in-Human Phase I Clinical Trial of an SFV-Based RNA Replicon Cancer Vaccine against HPV-Induced Cancers. Mol. Ther..

[69] Blakney A.K., Ip S., Geall A.J. (2021). An Update on Self-Amplifying MRNA Vaccine Development. Vaccines.

[70] Yang L., Tang L., Zhang M., Liu C. (2022). Recent Advances in the Molecular Design and Delivery Technology of MRNA for Vaccination Against Infectious Diseases. Front. Immunol..

[71] Knezevic I., Liu M.A., Peden K., Zhou T., Kang H.-N. (2021). Development of MRNA Vaccines: Scientific and Regulatory Issues. Vaccines.

[72] Jeeva S., Kim K.-H., Shin C.H., Wang B.-Z., Kang S.-M. (2021). An Update on MRNA-Based Viral Vaccines. Vaccines.

[73] Iavarone C., O’hagan D.T., Yu D., Delahaye N.F., Ulmer J.B. (2017). Mechanism of Action of MRNA-Based Vaccines. Expert Rev. Vaccines.

[74] Wallis J., Shenton D.P., Carlisle R.C. (2019). Novel Approaches for the Design, Delivery and Administration of Vaccine Technologies. Clin. Exp. Immunol..

[75] Wadhwa A., Aljabbari A., Lokras A., Foged C., Thakur A. (2020). Opportunities and Challenges in the Delivery of MRNA-Based Vaccines. Pharmaceutics.

[76] Liang Z., Zhu H., Wang X., Jing B., Li Z., Xia X., Sun H., Yang Y., Zhang W., Shi L. (2020). Adjuvants for Coronavirus Vaccines. Front. Immunol..

[77] Pardi N., Hogan M.J., Porter F.W., Weissman D. (2018). MRNA Vaccines—A New Era in Vaccinology. Nat. Rev. Drug Discov..

[78] Mascola J.R., Fauci A.S. (2020). Novel Vaccine Technologies for the 21st Century. Nat. Rev. Immunol..

[79] Pollard A.J., Bijker E.M. (2021). A Guide to Vaccinology: From Basic Principles to New Developments. Nat. Rev. Immunol..

[80] Coria L.M., Saposnik L.M., Pueblas Castro C., Castro E.F., Bruno L.A., Stone W.B., Pérez P.S., Darriba M.L., Chemes L.B., Alcain J. (2022). A Novel Bacterial Protease Inhibitor Adjuvant in RBD-Based COVID-19 Vaccine Formulations Containing Alum Increases Neutralizing Antibodies, Specific Germinal Center B Cells and Confers Protection Against SARS-CoV-2 Infection in Mice. Front. Immunol..

[81] Zhou Z., Zhang X., Li Q., Fu L., Wang M., Liu S., Wu J., Nie J., Zhang L., Zhao C. (2021). Unmethylated CpG Motif-Containing Genomic DNA Fragments of Bacillus Calmette-Guerin Improves Immune Response towards a DNA Vaccine for COVID-19. Vaccine.

[82] Nicholls E.F., Madera L., Hancock R.E.W. (2010). Immunomodulators as Adjuvants for Vaccines and Antimicrobial Therapy: Adjuvants for Vaccines and Antimicrobial Therapy. Ann. N. Y. Acad. Sci..

[83] Awate S., Babiuk L.A., Mutwiri G. (2013). Mechanisms of Action of Adjuvants. Front. Immunol..

[84] Li Q., Li Z., Deng N., Ding F., Li Y., Cai H. (2022). Built-in Adjuvants for Use in Vaccines. Eur. J. Med. Chem..

[85] Shi S., Zhu H., Xia X., Liang Z., Ma X., Sun B. (2019). Vaccine Adjuvants: Understanding the Structure and Mechanism of Adjuvanticity. Vaccine.

[86] Zhang N., Li K., Liu Z., Nandakumar K.S., Jiang S. (2022). A Perspective on the Roles of Adjuvants in Developing Highly Potent COVID-19 Vaccines. Viruses.

[87] Bonam S.R., Kotla N.G., Bohara R.A., Rochev Y., Webster T.J., Bayry J. (2021). Potential Immuno-Nanomedicine Strategies to Fight COVID-19 like Pulmonary Infections. Nano Today.

[88] Ciabattini A., Pettini E., Fiorino F., Pastore G., Andersen P., Pozzi G., Medaglini D. (2016). Modulation of Primary Immune Response by Different Vaccine Adjuvants. Front. Immunol..

[89] Nanishi E., Dowling D.J., Levy O. (2020). Toward Precision Adjuvants: Optimizing Science and Safety. Curr. Opin. Pediatr..

[90] Bonam S.R., Partidos C.D., Halmuthur S.K.M., Muller S. (2017). An Overview of Novel Adjuvants Designed for Improving Vaccine Efficacy. Trends Pharmacol. Sci..

[91] Korsholm K.S., Petersen R.V., Agger E.M., Andersen P. (2010). T-helper 1 and T-helper 2 Adjuvants Induce Distinct Differences in the Magnitude, Quality and Kinetics of the Early Inflammatory Response at the Site of Injection. Immunology.

[92] Zhu X., Zhu J. (2020). CD4 T Helper Cell Subsets and Related Human Immunological Disorders. Int. J. Mol. Sci..

[93] de Jong A., van Poelgeest M.I.E., van der Hulst J.M., Drijfhout J.W., Fleuren G.J., Melief C.J.M., Kenter G., Offringa R., van der Burg S.H. (2004). Human Papillomavirus Type 16-Positive Cervical Cancer Is Associated with Impaired CD4+ T-Cell Immunity against Early Antigens E2 and E6. Cancer Res..

[94] Elmusrati A., Wang J., Wang C.-Y. (2021). Tumor Microenvironment and Immune Evasion in Head and Neck Squamous Cell Carcinoma. Int. J. Oral. Sci..

[95] Welters M.J.P., Ma W., Santegoets S.J., Goedemans R., Ehsan I., Jordanova E.S., van Ham V.J., van Unen V., Koning F., van Egmond S.I. (2018). Intratumoral HPV16-Specific T Cells Constitute a Type I–Oriented Tumor Microenvironment to Improve Survival in HPV16-Driven Oropharyngeal Cancer. Clin. Cancer Res..

[96] Rocamora-Reverte L., Melzer F.L., Würzner R., Weinberger B. (2021). The Complex Role of Regulatory T Cells in Immunity and Aging. Front. Immunol..

[97] Goswami T.K., Singh M., Dhawan M., Mitra S., Emran T.B., Rabaan A.A., Mutair A.A., Alawi Z.A., Alhumaid S., Dhama K. (2022). Regulatory T Cells (Tregs) and Their Therapeutic Potential against Autoimmune Disorders–Advances and Challenges. Hum. Vaccines Immunother..

[98] Adurthi S., Krishna S., Mukherjee G., Bafna U.D., Devi U., Jayshree R.S. (2008). Original article: Regulatory T Cells in a Spectrum of HPV-Induced Cervical Lesions: Cervicitis, Cervical Intraepithelial Neoplasia and Squamous Cell Carcinoma: Regulatory T cells in cervical cancer. Am. J. Reprod. Immunol..

[99] Heeren A.M., Koster B.D., Samuels S., Ferns D.M., Chondronasiou D., Kenter G.G., Jordanova E.S., de Gruijl T.D. (2015). High and Interrelated Rates of PD-L1+CD14+ Antigen-Presenting Cells and Regulatory T Cells Mark the Microenvironment of Metastatic Lymph Nodes from Patients with Cervical Cancer. Cancer Immunol. Res..

[100] Seminerio I., Descamps G., Dupont S., de Marrez L., Laigle J.-A., Lechien J., Kindt N., Journe F., Saussez S. (2019). Infiltration of FoxP3+ Regulatory T Cells Is a Strong and Independent Prognostic Factor in Head and Neck Squamous Cell Carcinoma. Cancers.

[101] Karimi H., Soleimanjahi H., Abdoli A., Banijamali R.S. (2020). Combination Therapy Using Human Papillomavirus L1/E6/E7 Genes and Archaeosome: A Nanovaccine Confer Immuneadjuvanting Effects to Fight Cervical Cancer. Sci. Rep..

[102] HogenEsch H., O’Hagan D.T., Fox C.B. (2018). Optimizing the Utilization of Aluminum Adjuvants in Vaccines: You Might Just Get What You Want. npj Vaccines.

[103] Mann C.C.O., Hornung V. (2021). Molecular Mechanisms of Nonself Nucleic Acid Recognition by the Innate Immune System. Eur. J. Immunol..

[104] Schlee M., Hartmann G. (2016). Discriminating Self from Non-Self in Nucleic Acid Sensing. Nat. Rev. Immunol..

[105] Morelli M.P., Del Medico Zajac M.P., Pellegrini J.M., Amiano N.O., Tateosian N.L., Calamante G., Gherardi M.M., García V.E. (2020). IL-12 DNA Displays Efficient Adjuvant Effects Improving Immunogenicity of Ag85A in DNA Prime/MVA Boost Immunizations. Front. Cell. Infect. Microbiol..

[106] Freund I., Eigenbrod T., Helm M., Dalpke A. (2019). RNA Modifications Modulate Activation of Innate Toll-Like Receptors. Genes.

[107] Kobiyama K., Ishii K.J. (2022). Making Innate Sense of MRNA Vaccine Adjuvanticity. Nat. Immunol..

[108] Dülmen M., Muthmann N., Rentmeister A. (2021). Chemo-Enzymatic Modification of the 5’ Cap Maintains Translation and Increases Immunogenic Properties of MRNA. Angew. Chem. Int. Ed..

[109] Karikó K., Buckstein M., Ni H., Weissman D. (2005). Suppression of RNA Recognition by Toll-like Receptors: The Impact of Nucleoside Modification and the Evolutionary Origin of RNA. Immunity.

[110] Karikó K., Muramatsu H., Welsh F.A., Ludwig J., Kato H., Akira S., Weissman D. (2008). Incorporation of Pseudouridine Into MRNA Yields Superior Nonimmunogenic Vector with Increased Translational Capacity and Biological Stability. Mol. Ther..

[111] Abbasi S., Uchida S. (2021). Multifunctional Immunoadjuvants for Use in Minimalist Nucleic Acid Vaccines. Pharmaceutics.

[112] Liang Y., Huang L., Liu T. (2021). Development and Delivery Systems of MRNA Vaccines. Front. Bioeng. Biotechnol..

[113] Wang W., Saeed M., Zhou Y., Yang L., Wang D., Yu H. (2019). Non-viral Gene Delivery for Cancer Immunotherapy. J. Gene Med..

[114] Chen G., Zhao B., Ruiz E.F., Zhang F. (2022). Advances in the Polymeric Delivery of Nucleic Acid Vaccines. Theranostics.

[115] Torres-Vanegas J.D., Cruz J.C., Reyes L.H. (2021). Delivery Systems for Nucleic Acids and Proteins: Barriers, Cell Capture Pathways and Nanocarriers. Pharmaceutics.

[116] Tizard I.R. (2021). Adjuvants and Adjuvanticity. Vaccines for Veterinarians.

[117] Andrianov A.K., Fuerst T.R. (2021). Immunopotentiating and Delivery Systems for HCV Vaccines. Viruses.

[118] Ding Y., Li Z., Jaklenec A., Hu Q. (2021). Vaccine Delivery Systems toward Lymph Nodes. Adv. Drug Deliv. Rev..

[119] Aldosari B.N., Alfagih I.M., Almurshedi A.S. (2021). Lipid Nanoparticles as Delivery Systems for RNA-Based Vaccines. Pharmaceutics.

[120] Sahin U., Muik A., Derhovanessian E., Vogler I., Kranz L.M., Vormehr M., Baum A., Pascal K., Quandt J., Maurus D. (2020). COVID-19 Vaccine BNT162b1 Elicits Human Antibody and TH1 T Cell Responses. Nature.

[121] Hasan T., Kawanishi R., Akita H., Nishikawa Y. (2021). Toxoplasma Gondii GRA15 DNA Vaccine with a Liposomal Nanocarrier Composed of an SS-Cleavable and PH-Activated Lipid-like Material Induces Protective Immunity against Toxoplasmosis in Mice. Vaccines.

[122] Tian M., Zhou Z., Tan S., Fan X., Li L., Ullah N. (2018). Formulation in DDA-MPLA-TDB Liposome Enhances the Immunogenicity and Protective Efficacy of a DNA Vaccine against Mycobacterium Tuberculosis Infection. Front. Immunol..

[123] Zhao Z., Ma X., Zhang R., Hu F., Zhang T., Liu Y., Han M.H., You F., Yang Y., Zheng W. (2021). A Novel Liposome-Polymer Hybrid Nanoparticles Delivering a Multi-Epitope Self-Replication DNA Vaccine and Its Preliminary Immune Evaluation in Experimental Animals. Nanomed. Nanotechnol. Biol. Med..

[124] Peletta A., Prompetchara E., Tharakhet K., Kaewpang P., Buranapraditkun S., Techawiwattanaboon T., Jbilou T., Krangvichian P., Sirivichayakul S., Manopwisedjaroen S. (2021). DNA Vaccine Administered by Cationic Lipoplexes or by In Vivo Electroporation Induces Comparable Antibody Responses against SARS-CoV-2 in Mice. Vaccines.

[125] Perche F., Clemençon R., Schulze K., Ebensen T., Guzmán C.A., Pichon C. (2019). Neutral Lipopolyplexes for In Vivo Delivery of Conventional and Replicative RNA Vaccine. Mol. Ther. Nucleic Acids.

[126] Hosseinipour M.C., Innes C., Naidoo S., Mann P., Hutter J., Ramjee G., Sebe M., Maganga L., Herce M.E., deCamp A.C. (2020). Phase 1 Human Immunodeficiency Virus (HIV) Vaccine Trial to Evaluate the Safety and Immunogenicity of HIV Subtype C DNA and MF59-Adjuvanted Subtype C Envelope Protein. Clin. Infect. Dis..

[127] Schuh R.S., Bidone J., Poletto E., Pinheiro C.V., Pasqualim G., de Carvalho T.G., Farinon M., da Silva Diel D., Xavier R.M., Baldo G. (2018). Nasal Administration of Cationic Nanoemulsions as Nucleic Acids Delivery Systems Aiming at Mucopolysaccharidosis Type I Gene Therapy. Pharm. Res..

[128] Baden L.R., El Sahly H.M., Essink B., Kotloff K., Frey S., Novak R., Diemert D., Spector S.A., Rouphael N., Creech C.B. (2021). Efficacy and Safety of the MRNA-1273 SARS-CoV-2 Vaccine. N. Engl. J. Med..

[129] Polack F.P., Thomas S.J., Kitchin N., Absalon J., Gurtman A., Lockhart S., Perez J.L., Pérez Marc G., Moreira E.D., Zerbini C. (2020). Safety and Efficacy of the BNT162b2 MRNA COVID-19 Vaccine. N. Engl. J. Med..

[130] Mucker E.M., Karmali P.P., Vega J., Kwilas S.A., Wu H., Joselyn M., Ballantyne J., Sampey D., Mukthavaram R., Sullivan E. (2020). Lipid Nanoparticle Formulation Increases Efficiency of DNA-Vectored Vaccines/Immunoprophylaxis in Animals Including Transchromosomic Bovines. Sci. Rep..

[131] Besumbes E.S., Fornaguera C., Monge M., García-Celma M.J., Carrión J., Solans C., Dols-Perez A. (2019). PLGA Cationic Nanoparticles, Obtained from Nano-Emulsion Templating, as Potential DNA Vaccines. Eur. Polym. J..

[132] Soares E., Cordeiro R., Faneca H., Borges O. (2019). Polymeric Nanoengineered HBsAg DNA Vaccine Designed in Combination with Β-glucan. Int. J. Biol. Macromol..

[133] Hraber P., Bradfute S., Clarke E., Ye C., Pitard B. (2018). Amphiphilic Block Copolymer Delivery of a DNA Vaccine against Zika Virus. Vaccine.

[134] Zhao K., Rong G., Teng Q., Li X., Lan H., Yu L., Yu S., Jin Z., Chen G., Li Z. (2020). Dendrigraft Poly-L-Lysines Delivery of DNA Vaccine Effectively Enhances the Immunogenic Responses against H9N2 Avian Influenza Virus Infection in Chickens. Nanomed. Nanotechnol. Biol. Med..

[135] Ren J., Cao Y., Li L., Wang X., Lu H., Yang J., Wang S. (2021). Self-Assembled Polymeric Micelle as a Novel MRNA Delivery Carrier. J. Control. Release.

[136] Huang J., Jia R., Shen H., Wang M., Zhu D., Chen S., Liu M., Zhao X., Wu Y., Yang Q. (2018). Oral Delivery of a DNA Vaccine Expressing the PrM and E Genes: A Promising Vaccine Strategy against Flavivirus in Ducks. Sci. Rep..

[137] Kim B.-J., Jeong H., Seo H., Lee M.-H., Shin H.M., Kim B.-J. (2021). Recombinant Mycobacterium Paragordonae Expressing SARS-CoV-2 Receptor-Binding Domain as a Vaccine Candidate Against SARS-CoV-2 Infections. Front. Immunol..

[138] Liu D., Lu S., Zhang L., Ji M., Liu S., Wang S., Liu R. (2018). An Indoleamine 2, 3-Dioxygenase SiRNA Nanoparticle-Coated and Trp2-Displayed Recombinant Yeast Vaccine Inhibits Melanoma Tumor Growth in Mice. J. Control. Release.

[139] Han B., Xu K., Liu Z., Ge W., Shao S., Li P., Yan N., Li X., Zhang Z. (2019). Oral Yeast-Based DNA Vaccine Confers Effective Protection from Aeromonas Hydrophila Infection on Carassius Auratus. Fish Shellfish Immunol..

[140] Zakria H.M., Han B., Yue F., Mu L., Fang Y., Li X., Xu K., Zhang Z. (2019). Significant Body Mass Increase by Oral Administration of a Cascade of ShIL21-MSTN Yeast-Based DNA Vaccine in Mice. Biomed. Pharmacother..

[141] Zhang L., Peng H., Feng M., Zhang W., Li Y. (2021). Yeast Microcapsule-Mediated Oral Delivery of IL-1β ShRNA for Post-Traumatic Osteoarthritis Therapy. Mol. Ther. Nucleic Acids.

[142] Yan Y., Liu X.-Y., Lu A., Wang X.-Y., Jiang L.-X., Wang J.-C. (2022). Non-Viral Vectors for RNA Delivery. J. Control. Release.

[143] Mukai H., Ogawa K., Kato N., Kawakami S. (2022). Recent Advances in Lipid Nanoparticles for Delivery of Nucleic Acid, MRNA, and Gene Editing-Based Therapeutics. Drug Metab. Pharmacokinet..

[144] Fan Y., Marioli M., Zhang K. (2021). Analytical Characterization of Liposomes and Other Lipid Nanoparticles for Drug Delivery. J. Pharm. Biomed. Anal..

[145] Hou X., Zaks T., Langer R., Dong Y. (2021). Lipid Nanoparticles for MRNA Delivery. Nat. Rev. Mater..

[146] Filipczak N., Pan J., Yalamarty S.S.K., Torchilin V.P. (2020). Recent Advancements in Liposome Technology. Adv. Drug Deliv. Rev..

[147] Tenchov R., Bird R., Curtze A.E., Zhou Q. (2021). Lipid Nanoparticles—From Liposomes to MRNA Vaccine Delivery, a Landscape of Research Diversity and Advancement. ACS Nano.

[148] Tretiakova D.S., Vodovozova E.L. (2022). Liposomes as Adjuvants and Vaccine Delivery Systems. Biochem. Moscow Suppl. Ser. A.

[149] Ponti F., Campolungo M., Melchiori C., Bono N., Candiani G. (2021). Cationic Lipids for Gene Delivery: Many Players, One Goal. Chem. Phys. Lipids.

[150] Wang N., Chen M., Wang T. (2019). Liposomes Used as a Vaccine Adjuvant-Delivery System: From Basics to Clinical Immunization. J. Control. Release.

[151] Pal Singh P., Vithalapuram V., Metre S., Kodipyaka R. (2020). Lipoplex-Based Therapeutics for Effective Oligonucleotide Delivery: A Compendious Review. J. Liposome Res..

[152] Berger M., Lechanteur A., Evrard B., Piel G. (2021). Innovative Lipoplexes Formulations with Enhanced SiRNA Efficacy for Cancer Treatment: Where Are We Now?. Int. J. Pharm..

[153] Yonezawa S., Koide H., Asai T. (2020). Recent Advances in SiRNA Delivery Mediated by Lipid-Based Nanoparticles. Adv. Drug Deliv. Rev..

[154] Ko E.-J., Kang S.-M. (2018). Immunology and Efficacy of MF59-Adjuvanted Vaccines. Hum. Vaccines Immunother..

[155] Zhang C., Ma Y., Zhang J., Kuo J.C.-T., Zhang Z., Xie H., Zhu J., Liu T. (2022). Modification of Lipid-Based Nanoparticles: An Efficient Delivery System for Nucleic Acid-Based Immunotherapy. Molecules.

[156] Teixeira H.F., Bruxel F., Fraga M., Schuh R.S., Zorzi G.K., Matte U., Fattal E. (2017). Cationic Nanoemulsions as Nucleic Acids Delivery Systems. Int. J. Pharm..

[157] Schuh R.S., Poletto É., Fachel F.N.S., Matte U., Baldo G., Teixeira H.F. (2018). Physicochemical Properties of Cationic Nanoemulsions and Liposomes Obtained by Microfluidization Complexed with a Single Plasmid or along with an Oligonucleotide: Implications for CRISPR/Cas Technology. J. Colloid Interface Sci..

[158] Samaridou E., Heyes J., Lutwyche P. (2020). Lipid Nanoparticles for Nucleic Acid Delivery: Current Perspectives. Adv. Drug Deliv. Rev..

[159] Thi T.T.H., Suys E.J.A., Lee J.S., Nguyen D.H., Park K.D., Truong N.P. (2021). Lipid-Based Nanoparticles in the Clinic and Clinical Trials: From Cancer Nanomedicine to COVID-19 Vaccines. Vaccines.

[160] Teijaro J.R., Farber D.L. (2021). COVID-19 Vaccines: Modes of Immune Activation and Future Challenges. Nat. Rev. Immunol..

[161] Schoenmaker L., Witzigmann D., Kulkarni J.A., Verbeke R., Kersten G., Jiskoot W., Crommelin D.J.A. (2021). MRNA-Lipid Nanoparticle COVID-19 Vaccines: Structure and Stability. Int. J. Pharm..

[162] Fahrni M.L., Ismail I.A.-N., Refi D.M., Almeman A., Yaakob N.C., Saman K.M., Mansor N.F., Noordin N., Babar Z.-U.-D. (2022). Management of COVID-19 Vaccines Cold Chain Logistics: A Scoping Review. J. Pharm. Policy Pract..

[163] Wang J.-L., Hanafy M.S., Xu H., Leal J., Zhai Y., Ghosh D., Robert O.W., Smyth H.D.C., Cui Z. (2021). Aerosolizable SiRNA-Encapsulated Solid Lipid Nanoparticles Prepared by Thin-Film Freeze-Drying for Potential Pulmonary Delivery. Int. J. Pharm..

[164] van den Berg A.I.S., Yun C.-O., Schiffelers R.M., Hennink W.E. (2021). Polymeric Delivery Systems for Nucleic Acid Therapeutics: Approaching the Clinic. J. Control. Release.

[165] Grun M.K., Suberi A., Shin K., Lee T., Gomerdinger V., Moscato Z.M., Piotrowski-Daspit A.S., Saltzman W.M. (2021). PEGylation of Poly(Amine-Co-Ester) Polyplexes for Tunable Gene Delivery. Biomaterials.

[166] Shiraishi K., Yokoyama M. (2019). Toxicity and Immunogenicity Concerns Related to PEGylated-Micelle Carrier Systems: A Review. Sci. Technol. Adv. Mater..

[167] Su S., Kang P.M. (2020). Systemic Review of Biodegradable Nanomaterials in Nanomedicine. Nanomaterials.

[168] Ita K. (2020). Polyplexes for Gene and Nucleic Acid Delivery: Progress and Bottlenecks. Eur. J. Pharm. Sci..

[169] Makadia H.K., Siegel S.J. (2011). Poly Lactic-Co-Glycolic Acid (PLGA) as Biodegradable Controlled Drug Delivery Carrier. Polymers.

[170] Lu H., Cai J., Zhang K. (2021). Synthetic Approaches for Copolymers Containing Nucleic Acids and Analogues: Challenges and Opportunities. Polym. Chem..

[171] Bose R.J., Kim M., Chang J.H., Paulmurugan R., Moon J.J., Koh W.-G., Lee S.-H., Park H. (2019). Biodegradable Polymers for Modern Vaccine Development. J. Ind. Eng. Chem..

[172] Mittal P., Saharan A., Verma R., Altalbawy F.M.A., Alfaidi M.A., Batiha G.E.-S., Akter W., Gautam R.K., Uddin M., Rahman M. (2021). Dendrimers: A New Race of Pharmaceutical Nanocarriers. BioMed Res. Int..

[173] Abedi-Gaballu F., Dehghan G., Ghaffari M., Yekta R., Abbaspour-Ravasjani S., Baradaran B., Dolatabadi J.E.N., Hamblin M.R. (2018). PAMAM Dendrimers as Efficient Drug and Gene Delivery Nanosystems for Cancer Therapy. Appl. Mater. Today.

[174] Ghezzi M., Pescina S., Padula C., Santi P., Del Favero E., Cantù L., Nicoli S. (2021). Polymeric Micelles in Drug Delivery: An Insight of the Techniques for Their Characterization and Assessment in Biorelevant Conditions. J. Control. Release.

[175] Ou B.S., Saouaf O.M., Baillet J., Appel E.A. (2022). Sustained Delivery Approaches to Improving Adaptive Immune Responses. Adv. Drug Deliv. Rev..

[176] Yurina V. (2018). Live Bacterial Vectors—A Promising DNA Vaccine Delivery System. Med. Sci..

[177] Asadi K., Gholami A. (2021). Virosome-Based Nanovaccines; a Promising Bioinspiration and Biomimetic Approach for Preventing Viral Diseases: A Review. Int. J. Biol. Macromol..

[178] Silva A.J.D., de Macêdo L.S., Leal L.R.S., de Jesus A.L.S., Freitas A.C. (2021). Yeasts as a Promising Delivery Platform for DNA and RNA Vaccines. FEMS Yeast Res..

[179] Tan Y., Chen L., Li K., Lou B., Liu Y., Liu Z. (2022). Yeast as Carrier for Drug Delivery and Vaccine Construction. J. Control. Release.

[180] Ramamoorth M. (2015). Non Viral Vectors in Gene Therapy—An Overview. J. Clin. Diagn. Res..

[181] Zhu F.-C., Li Y.-H., Guan X.-H., Hou L.-H., Wang W.-J., Li J.-X., Wu S.-P., Wang B.-S., Wang Z., Wang L. (2020). Safety, Tolerability, and Immunogenicity of a Recombinant Adenovirus Type-5 Vectored COVID-19 Vaccine: A Dose-Escalation, Open-Label, Non-Randomised, First-in-Human Trial. Lancet.

[182] Kheiri M.T., Jamali A., Shenagari M., Hashemi H., Sabahi F., Atyabi F., Saghiri R. (2012). Influenza Virosome/DNA Vaccine Complex as a New Formulation to Induce Intra-Subtypic Protection against Influenza Virus Challenge. Antivir. Res..

[183] Fan J., Jin S., Gilmartin L., Toth I., Hussein W.M., Stephenson R.J. (2022). Advances in Infectious Disease Vaccine Adjuvants. Vaccines.

[184] Loo Y.S., Bose R.J., McCarthy J.R., Azmi I.D.M., Madheswaran T. (2021). Biomimetic Bacterial and Viral-Based Nanovesicles for Drug Delivery, Theranostics, and Vaccine Applications. Drug Discov. Today.

[185] Alemzadeh E., Dehshahri A., Izadpanah K., Ahmadi F. (2018). Plant Virus Nanoparticles: Novel and Robust Nanocarriers for Drug Delivery and Imaging. Colloids Surf. B Biointerfaces.

[186] Beatty P.H., Lewis J.D. (2019). Cowpea Mosaic Virus Nanoparticles for Cancer Imaging and Therapy. Adv. Drug Deliv. Rev..

[187] Cao Z., Liu J. (2020). Bacteria and Bacterial Derivatives as Drug Carriers for Cancer Therapy. J. Control. Release.

[188] Long Q., Zheng P., Zheng X., Li W., Hua L., Yang Z., Huang W., Ma Y. (2022). Engineered Bacterial Membrane Vesicles Are Promising Carriers for Vaccine Design and Tumor Immunotherapy. Adv. Drug Deliv. Rev..

[189] Kazi T.A., Acharya A., Mukhopadhyay B.C., Mandal S., Arukha A.P., Nayak S., Biswas S.R. (2022). Plasmid-Based Gene Expression Systems for Lactic Acid Bacteria: A Review. Microorganisms.

[190] Kang S.-R., Nguyen D.-H., Yoo S.W., Min J.-J. (2022). Bacteria and Bacterial Derivatives as Delivery Carriers for Immunotherapy. Adv. Drug Deliv. Rev..

[191] Adamiak N., Krawczyk K.T., Locht C., Kowalewicz-Kulbat M. (2021). Archaeosomes and Gas Vesicles as Tools for Vaccine Development. Front. Immunol..

[192] Alexander E. (2021). Yeasts in Nanotechnology-Enabled Oral Vaccine and Gene Delivery. Bioengineered.

[193] Kumar R., Kumar P. (2019). Yeast-Based Vaccines: New Perspective in Vaccine Development and Application. FEMS Yeast Res..

[194] Duman-Scheel M. (2019). Saccharomyces Cerevisiae (Baker’s Yeast) as an Interfering RNA Expression and Delivery System. Curr. Drug Targets.

[195] Baranov M.V., Kumar M., Sacanna S., Thutupalli S., van den Bogaart G. (2021). Modulation of Immune Responses by Particle Size and Shape. Front. Immunol..

[196] Cai T., Liu H., Zhang S., Hu J., Zhang L. (2021). Delivery of Nanovaccine towards Lymphoid Organs: Recent Strategies in Enhancing Cancer Immunotherapy. J. Nanobiotechnol..

[197] Hampton H.R., Chtanova T. (2019). Lymphatic Migration of Immune Cells. Front. Immunol..

[198] Petkar K.C., Patil S.M., Chavhan S.S., Kaneko K., Sawant K.K., Kunda N.K., Saleem I.Y. (2021). An Overview of Nanocarrier-Based Adjuvants for Vaccine Delivery. Pharmaceutics.

[199] Blakney A.K., Yilmaz G., McKay P.F., Becer C.R., Shattock R.J. (2018). One Size Does Not Fit All: The Effect of Chain Length and Charge Density of Poly(Ethylene Imine) Based Copolymers on Delivery of PDNA, MRNA, and RepRNA Polyplexes. Biomacromolecules.

[200] Kesharwani P., Gothwal A., Iyer A.K., Jain K., Chourasia M.K., Gupta U. (2018). Dendrimer Nanohybrid Carrier Systems: An Expanding Horizon for Targeted Drug and Gene Delivery. Drug Discov. Today.

[201] Mitchell M.J., Billingsley M.M., Haley R.M., Wechsler M.E., Peppas N.A., Langer R. (2021). Engineering Precision Nanoparticles for Drug Delivery. Nat. Rev. Drug Discov..

[202] Augustine R., Hasan A., Primavera R., Wilson R.J., Thakor A.S., Kevadiya B.D. (2020). Cellular Uptake and Retention of Nanoparticles: Insights on Particle Properties and Interaction with Cellular Components. Mater. Today Commun..

[203] Schudel A., Francis D.M., Thomas S.N. (2019). Material Design for Lymph Node Drug Delivery. Nat. Rev. Mater..

[204] Jiang Z., Thayumanavan S. (2020). Noncationic Material Design for Nucleic Acid Delivery. Adv. Ther..

[205] Trimble C.L., Morrow M.P., Kraynyak K.A., Shen X., Dallas M., Yan J., Edwards L., Parker R.L., Denny L., Giffear M. (2015). Safety, Efficacy, and Immunogenicity of VGX-3100, a Therapeutic Synthetic DNA Vaccine Targeting Human Papillomavirus 16 and 18 E6 and E7 Proteins for Cervical Intraepithelial Neoplasia 2/3: A Randomised, Double-Blind, Placebo-Controlled Phase 2b Trial. Lancet.

[206] Lee S.Y., Huang Z., Kang T.H., Soong R.-S., Knoff J., Axenfeld E., Wang C., Alvarez R.D., Chen C.-S., Hung C.-F. (2013). Histone Deacetylase Inhibitor AR-42 Enhances E7-Specific CD8+ T Cell-Mediated Antitumor Immunity Induced by Therapeutic HPV DNA Vaccination. J. Mol. Med..

[207] Garza-Morales R., Perez-Trujillo J., Martinez-Jaramillo E., Saucedo-Cardenas O., Loera-Arias M., Garcia-Garcia A., Rodriguez-Rocha H., Yolcu E., Shirwan H., Gomez-Gutierrez J. (2019). A DNA Vaccine Encoding SA-4-1BBL Fused to HPV-16 E7 Antigen Has Prophylactic and Therapeutic Efficacy in a Cervical Cancer Mouse Model. Cancers.

[208] Jazayeri S.D., Lim H.X., Shameli K., Yeap S.K., Poh C.L. (2021). Nano and Microparticles as Potential Oral Vaccine Carriers and Adjuvants Against Infectious Diseases. Front. Pharmacol..

